# HIV-1 phylodynamic analysis among people who inject drugs in Pakistan correlates with trends in illicit opioid trade

**DOI:** 10.1371/journal.pone.0237560

**Published:** 2020-08-28

**Authors:** François Cholette, Jeffrey Joy, Yann Pelcat, Laura H. Thompson, Richard Pilon, John Ho, Rupert Capina, Chris Archibald, James F. Blanchard, Faran Emmanuel, Tahira Reza, Nosheen Dar, Richard Harrigan, John Kim, Paul Sandstrom

**Affiliations:** 1 National HIV and Retrovirology Laboratories, National Microbiology Laboratory at the JC Wilt Infectious Diseases Research Centre, Public Health Agency of Canada, Winnipeg, Manitoba, Canada; 2 Department of Community Health Sciences, University of Manitoba, Winnipeg, Manitoba, Canada; 3 British Columbia Centre for Excellence in HIV/AIDS, Vancouver, British Columbia, Canada; 4 Department of Medicine, University of British Columbia, Vancouver, British Columbia, Canada; 5 Bioinformatics Programme, University of British Columbia, Vancouver, British Columbia, Canada; 6 Public Health Risk Sciences Division, National Microbiology Laboratory, Public Health Agency of Canada, Saint-Hyacinthe, Québec, Canada; 7 Centre for Communicable Diseases and Infection Control, Surveillance and Epidemiology Division, Public Health Agency of Canada, Ottawa, Ontario, Canada; 8 Centre for Global Public Health, University of Manitoba, Winnipeg, Canada; 9 Centre for Global Public Health, Pakistan, Chak Shahzad, Islamabad, Pakistan; 10 Canada-Pakistan HIV/AIDS Surveillance Project, Islamabad, Pakistan; 11 Medical Microbiology and Infectious Diseases, University of Manitoba, Winnipeg, Manitoba, Canada; University of Cincinnati College of Medicine, UNITED STATES

## Abstract

Pakistan is considered by the World Health Organization to currently have a “concentrated” HIV-1 epidemic due to a rapid rise in infections among people who inject drugs (PWID). Prevalence among the country’s nearly 105,000 PWID is estimated to be 37.8% but has been shown to be higher in several large urban centers. A lack of public health resources, the common use of professional injectors and unsafe injection practices are believed to have fueled the outbreak. Here we evaluate the molecular characteristics of HIV-1 sequences (n = 290) from PWID in several Pakistani cities to examine transmission dynamics and the association between rates of HIV-1 transmission with regards to regional trends in opioid trafficking. Tip-to-tip (patristic) distance based phylogenetic cluster inferences and BEAST2 Bayesian phylodynamic analyses of time-stamped data were performed on HIV-1 *pol* sequences generated from dried blood spots collected from 1,453 PWID as part of a cross-sectional survey conducted in Pakistan during 2014/2015. Overall, subtype A1 strains were dominant (75.2%) followed by CRF02_AG (14.1%), recombinants/unassigned (7.2%), CRF35_AD (2.1%), G (1.0%) and C (0.3%). Nearly three quarters of the PWID HIV-1 sequences belonged to one of five distinct phylogenetic clusters. Just below half (44.4%) of individuals in the largest cluster (n = 118) did seek help injecting from professional injectors which was previously identified as a strong correlate of HIV-1 infection. Spikes in estimated HIV-1 effective population sizes coincided with increases in opium poppy cultivation in Afghanistan, Pakistan’s western neighbor. Structured coalescent analysis was undertaken in order to investigate the spatial relationship of HIV-1 transmission among the various cities under study. In general terms, our analysis placed the city of Larkana at the center of the PWID HIV-1 epidemic in Pakistan which is consistent with previous epidemiological data.

## Introduction

Pakistan is considered to have transitioned from a “low prevalence, high risk” to a “concentrated” epidemic stage owing primarily to a rapid rise in infections among people who inject drugs (PWID, www.nacp.gov.pk/whatwedo/surveillance.html) [1]. While HIV-1 prevalence within the general population remains below 0.1%, prevalence among the country’s nearly 105,000 PWID is estimated to be 37.8% with even higher prevalence in selected urban centres (www.nacp.gov.pk/whatwedo/surveillance.html) [1, 2]. Perhaps more concerning is the significant rise in PWID associated HIV-1 prevalence over an extremely short interval that has been observed in certain cities, indicative of uncontrolled HIV-1 transmission resulting from a lack of public health resources. Concern remains that the HIV-1 epidemic within the country’s PWID population could spread to other at-risk populations such as male, transgender and female sex workers, thereby intensifying epidemics in high risk sexual networks in Pakistan.

We previously reported an explosive HIV-1 epidemic among PWID in the central Pakistani city of Sargodha in Punjab province [3]. Sequential cross-sectional surveys revealed a significant increase in HIV-1 prevalence within Sargodha’s PWID population from 9.0% in 2005–2006 to 51.5% in 2006–2007. The city is located along one of the country’s most important drug trafficking routes, connecting opioid production areas in eastern Afghanistan with southern and central Pakistan, ultimately providing the city’s estimated 2,500 PWID with easy access to inexpensive heroin (www.unodc.org/documents/wdr/WDR_2010/World_Drug_Report_2010_lo-res.pdf) [4]. Large sharing networks and unusual injection practices [5] which facilitate direct transfer of blood between individuals appear to be the main epidemic drivers [3]. The relationship between the HIV-1 outbreak in Sargodha and HIV-1 infections occurring in PWID or associated at risk populations in other regions of Pakistan remains unclear. In particular, a better understanding of the role that PWID migration between cities, for example as commercial truck drivers or migrant agricultural workers [6], plays in driving HIV-1 expansion will be critical for the development of sound and effective public health policies.

It has been reported that HIV-1 clade A1 infections in the Pakistani port city of Karachi may be the result of onward transmission among PWID of a highly similar founder virus [7]. Phylogenetic analysis of HIV-1 *gag* sequences derived from 26 HIV-1 positive PWID generated a single monophyletic clade A1 cluster with an inter-sequence identity of greater than 98%. In addition, Rai *et al*. (2010) deduced that the Karachi A1 HIV-1 epidemic was likely seeded by migrant contract workers who had been deported to Pakistan in the early 1990s after supposedly contracting HIV-1 through contact with sex workers in the Middle East. In this study, using HIV-1 sequence data from HIV-1 positive dried blood spot (DBS) specimens collected as part of a cross-sectional survey conducted in 2014, we sought to use phylodynamic methods to better understand the dynamics of HIV-1 transmission within Pakistan’s at risk populations. Specifically, to examine HIV-1 transmission clusters within and among cities in Pakistan and test hypotheses concerning the association between rates of HIV-1 transmission and regional trends in opioid trafficking.

## Results

### Serology, amplification and sequencing

Serological testing for HIV-1 determined that 367 of the 1,453 (25.6%) 2014 PWID survey participants were HIV-1 reactive while seventeen did not have sufficient specimen for testing. Amplification and sequencing of HIV-1 was attempted on all available HIV-1 serology reactive PWID specimens. In total, we were able to amplify 290 *pol* sequences of the 367 (79.0%) available specimens. In general, 80% of the HIV-1 reactive DBS specimens could be amplified from each city except Hyderabad where amplification success was closer to 70%. The observed 20–30% amplification failure was likely due to nucleic acid degradation during storage [8] and/or low viral loads (≦ 1,000 copies/mL) [9]. Even though our HIV-1 genotyping primers have been validated in-house as part of the Canadian HIV-1 Strain and Drug Resistance Surveillance program, we cannot rule out the possibility of amplification failure attributed to poor primer annealing efficiency to sequences from unique HIV-1 circulating recombinant forms (CRFs) recently reported in Pakistan [10].

### HIV-1 subtype distribution

**Fig 1:**
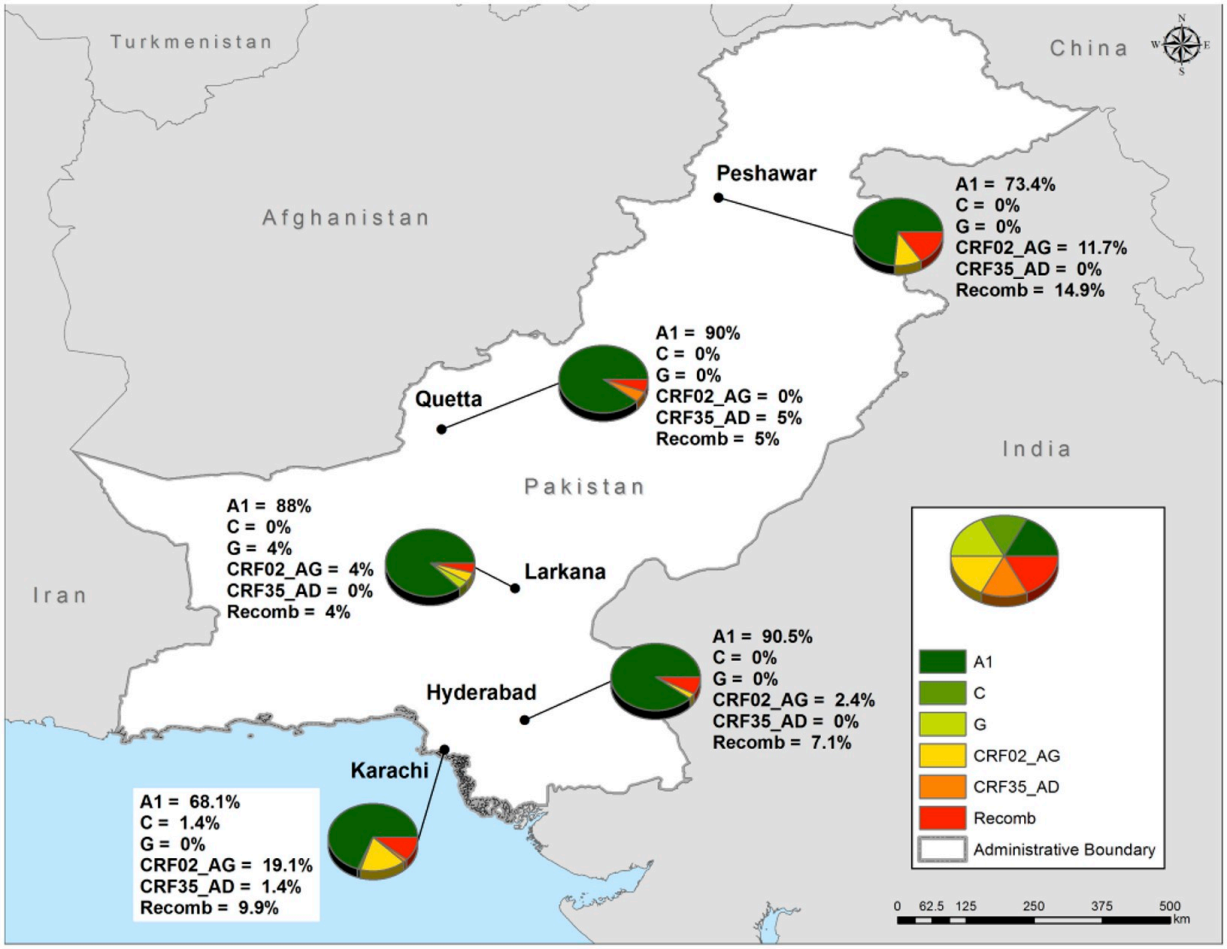
Geographic HIV-1 subtype distribution among people who inject drugs in Pakistan. “Recomb” refers to HIV-1 sequences that were unsuccessfully subtyped (i.e. labelled as recombinant and/or unassigned) by the automated subtyping tools. This map was created using ESRI ArcMap software version 10.5., using open source data from the Global Administrative Areas version 2.8 (https://gadm.org/data.html).

Each viral sequence from our study was assigned a subtype based on an analysis performed with automated HIV-1 subtyping tools (S1 Table). The geographic distribution of HIV-1 subtypes among PWID is depicted in Fig 1. Overall, subtype A1 strains were dominant (75.2%) followed by CRF02_AG (14.1%), recombinants/unassigned (7.2%), CRF35_AD (2.1%), G (1.0%) and C (0.3%).

### Phylogenetic clusters

**Fig 2:**
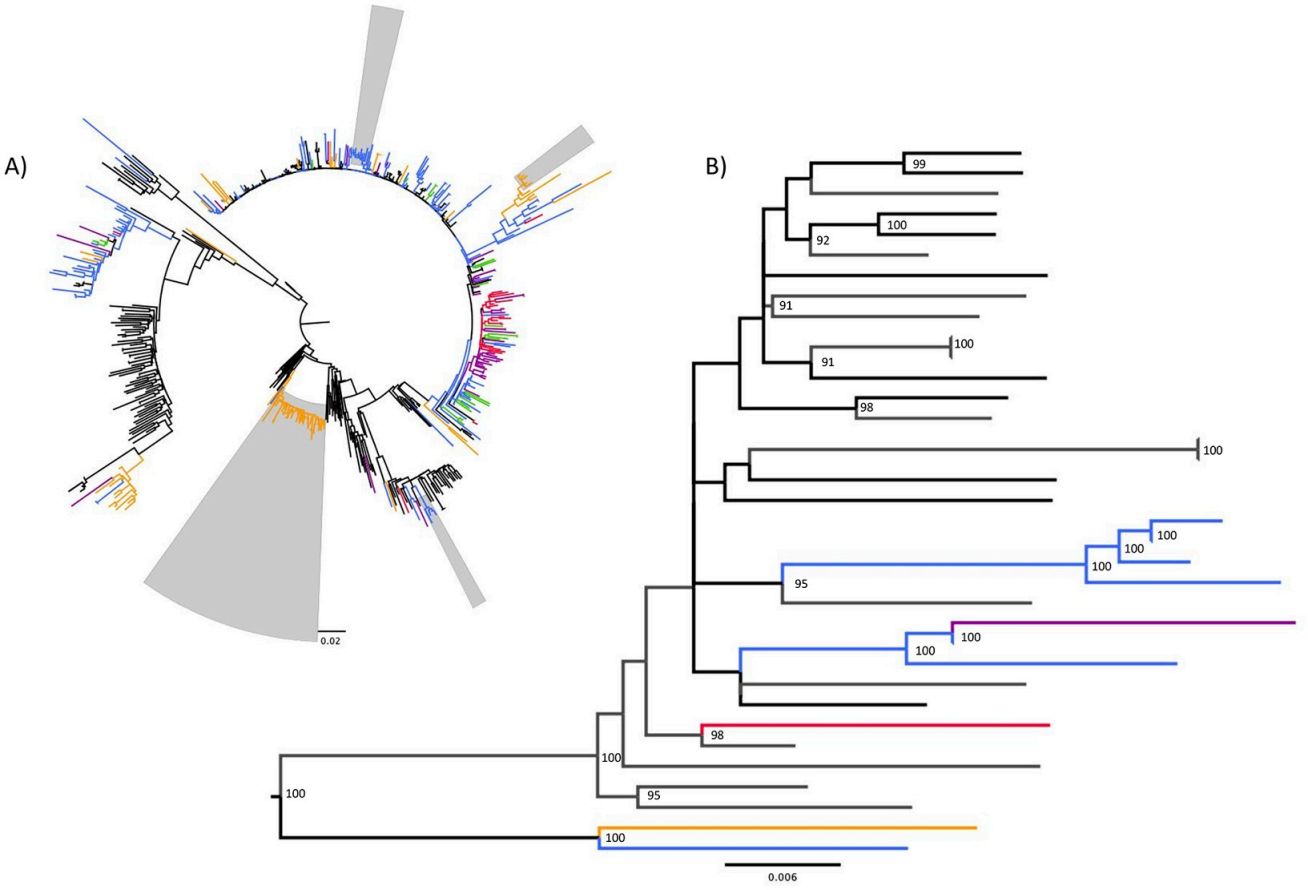
Maximum likelihood phylogenetic tree for HIV-1 *pol s*equences (n = 561). A) Viral strain sequences from people who inject drugs in Pakistan are annotated according to city of origin: Karachi (blue, n = 109 [19.4%]), Peshawar (orange, n = 94 [16.7%]), Hyderabad (purple, n = 42 [7.5%]), Larkana (green, n = 25 [4.5%]) and Quetta (red, n = 20 [3.6%]) while branches representing background sequences (n = 271 [48.3%]) were left black. Monophyletic clusters identified by Cluster Picker are highlighted by light grey bars. B) Sub-tree from principal maximum likelihood phylogenetic tree from HIV-1 *pol* sequences used for cluster analysis presenting HIV-1 subtype CRF35_AD sequences from people who inject drugs in Pakistan. The numbers on the branches represent the percentage of maximum likelihood ultra-fast bootstrap samples (100,000 replicates) where the node is supported by a value of ≥ 90%. Branches are annotated according to city or country of origin: Karachi (blue, n = 7 [20.0%]), Peshawar (orange, n = 1 [2.9%]), Hyderabad (purple, n = 1 [2.9%]), Quetta (red, n = 1 [2.9%]), Afghanistan (black, n = 10 [28.6%]), and grey (Iran, n = 15 [42.8%]).

The ML analysis of PWID sequences was done with background sequences consisting of all available HIV-1 *pol* sequences from Pakistan in the Los Alamos HIV database as well as closely related sequences determined by BLASTn [11] searches. Details regarding these sequences can be found in S2 Table. The phylogenetic tree inferred by ML analysis (Fig 2a) reveals close relationship between PWID infections, most notably in Peshawar. Of particular interest, several HIV-1 subtype CRF35_AD PWID sequences from our study were nested among sequences from Afghanistan and the Islamic Republic of Iran (Fig 2b), supported by a high bootstrap value, suggesting a close genetic relationship.

**Fig 3:**
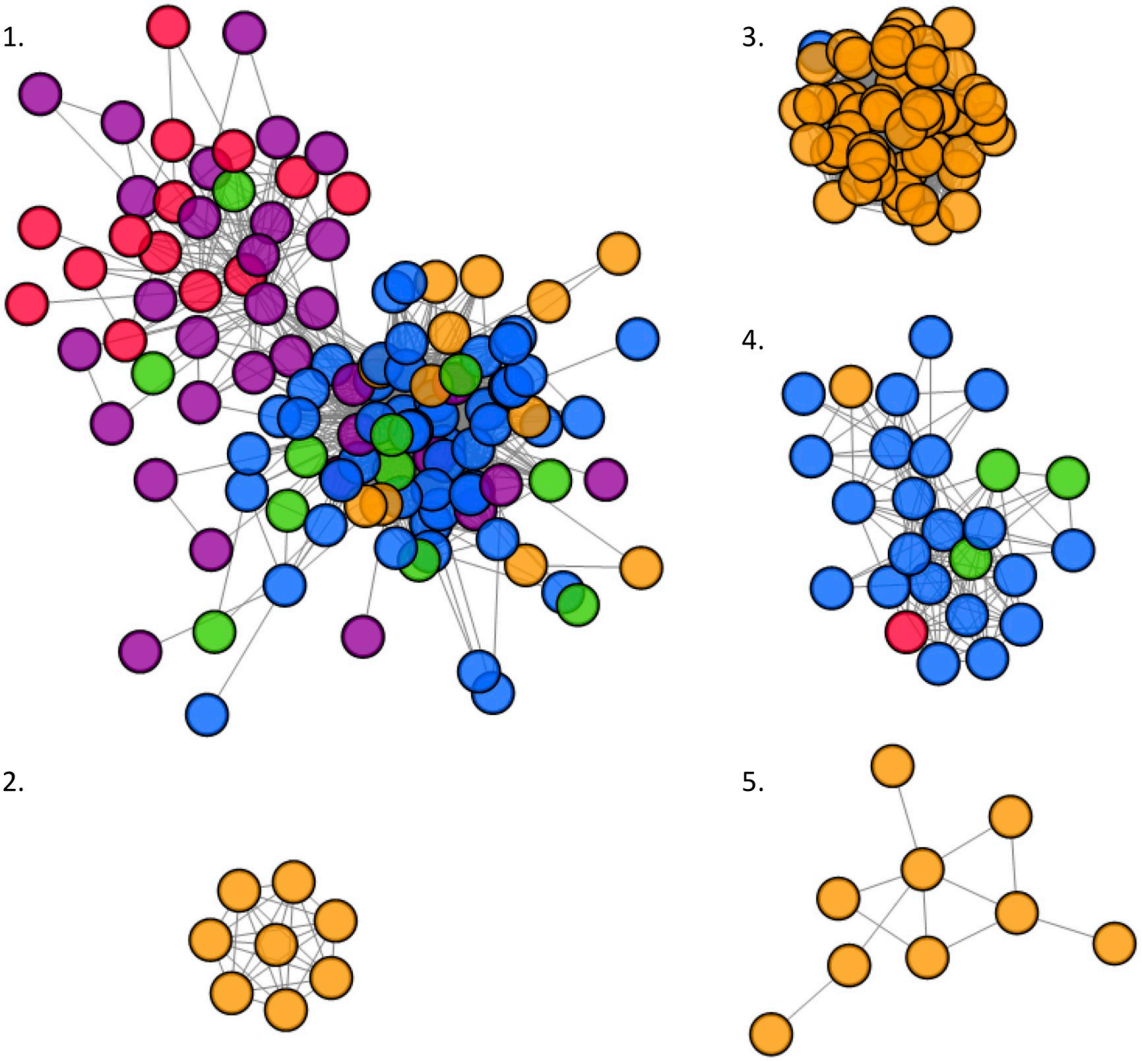
Phylogenetic clusters inferred using tip-to-tip (patristic) distance between sequences measured on phylogenetic trees. Viral strain sequences from people who inject drugs in Pakistan are annotated according to city of origin: Karachi (blue), Peshawar (orange), Hyderabad (purple), Larkana (green) and Quetta (red).

Out of the 290 HIV-1 sequences collected from PWIDs, 213 (73.5%) were associated with one of several phylogenetic clusters. In total, 5 distinct clusters ranging from 8 to 118 sequences in size were identified using tip-to-tip (patristic) distances (Table 1, Fig 3). Altogether, the odds of clustering among sequences from PWID who sought help injecting drugs was 10.1% (OR 0.899; 95% CI, 0.523–1.533) less compared to sequences from PWID who did not seek help injecting but this was found to be non-significant (X^2^ = 0.064, df = 1, p>0.05).

**Table 1:** Description of phylogenetic clusters identified by tip-to-tip (patristic) distances.

Cluster ID	Sequences (n =)	City					Help injecting		
		HYD (n =)	KAR (n =)	LAR (n =)	PES (n =)	QUE (n =)	Yes (n =)	No (n =)	N/A (n =)
1	118	32	49	11	12	14	52	65	1
2	8	-	-	-	8	-	2	6	-
3	52	-	1	-	51	-	12	40	-
4	26	-	21	3	1	1	10	16	-
5	9	-	-	-	9	-	4	5	-

Hyderabad, HYD; Karachi, KAR; Larkana, LAR; Peshawar, PES; Quetta, QUE; information not available, N/A

### HIV-1 phylodynamics

**Fig 4:**
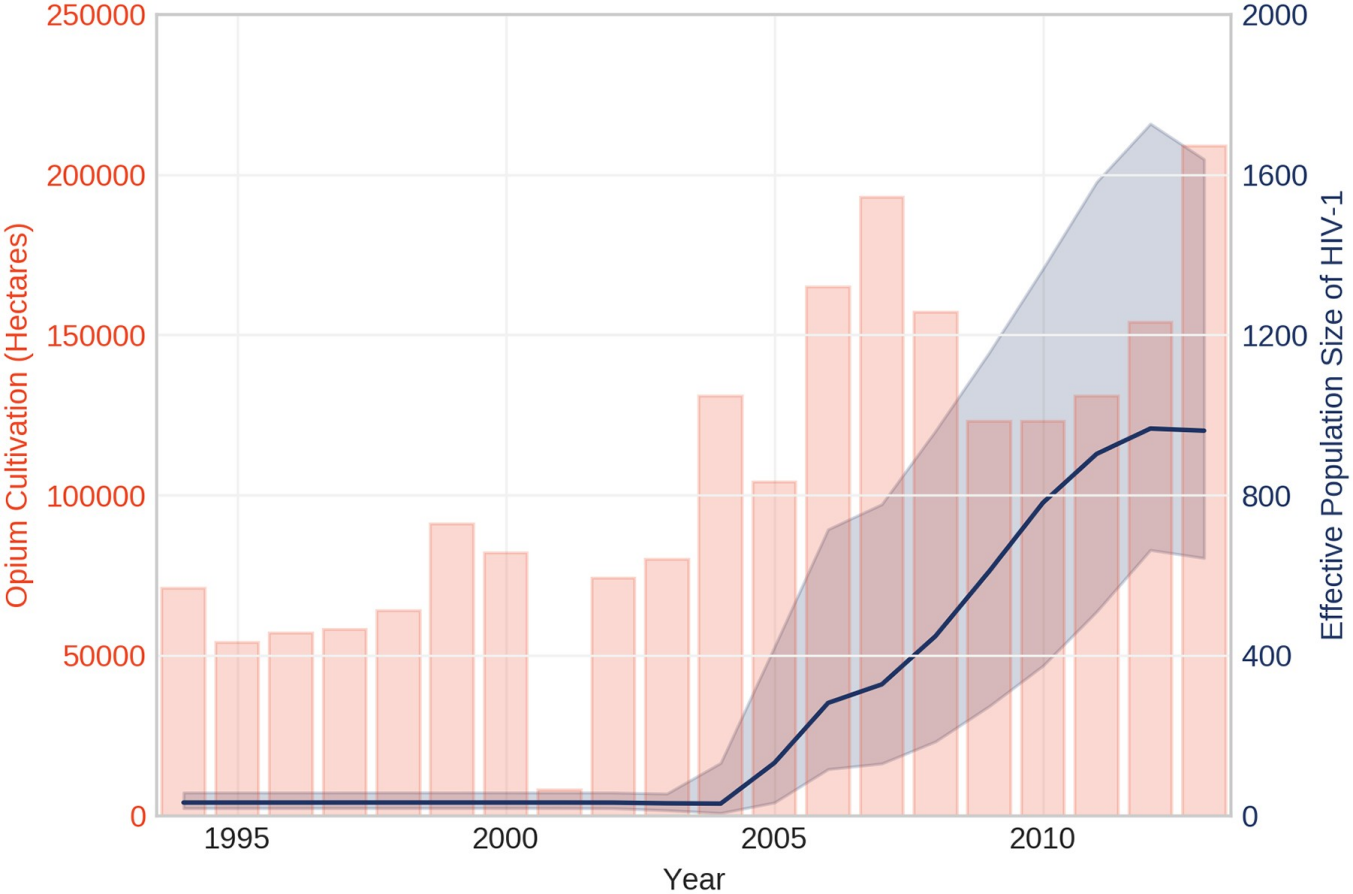
Effective population size estimates of HIV-1 among people who inject drugs in Pakistan. The median effective population size is shown by the solid blue line, with the 95% highest and lowest posterior densities represented by the area shaded in blue. The timeframe dates back to the early 1980’s but estimates up to 1994 only are displayed for legibility. Opium poppy cultivation in Afghanistan, according to estimates by the United Nations Office on Drugs and Crime [12], is represented by light orange bars.

Bayesian skyline plot analysis (Fig 4) for the period of 1994 to 2013 suggests that HIV-1 among PWID in Pakistan experienced an initial phase of exponential growth sometime around 2004 and peaked in 2006. Over the next two years, only minor changes to the median effective population size were observed until a second instance of exponential growth occurred around 2008 reaching a peak in 2012. Both spikes in HIV-1 median effective population size overlapped with increases in opium cultivation (Fig 4) in Pakistan’s neighboring country to the west, Afghanistan. Overall, there was a positive correlation between median HIV-1 effective population size and opium cultivation (r = 0.46, n = 19, p < 0.001).

**Fig 5:**
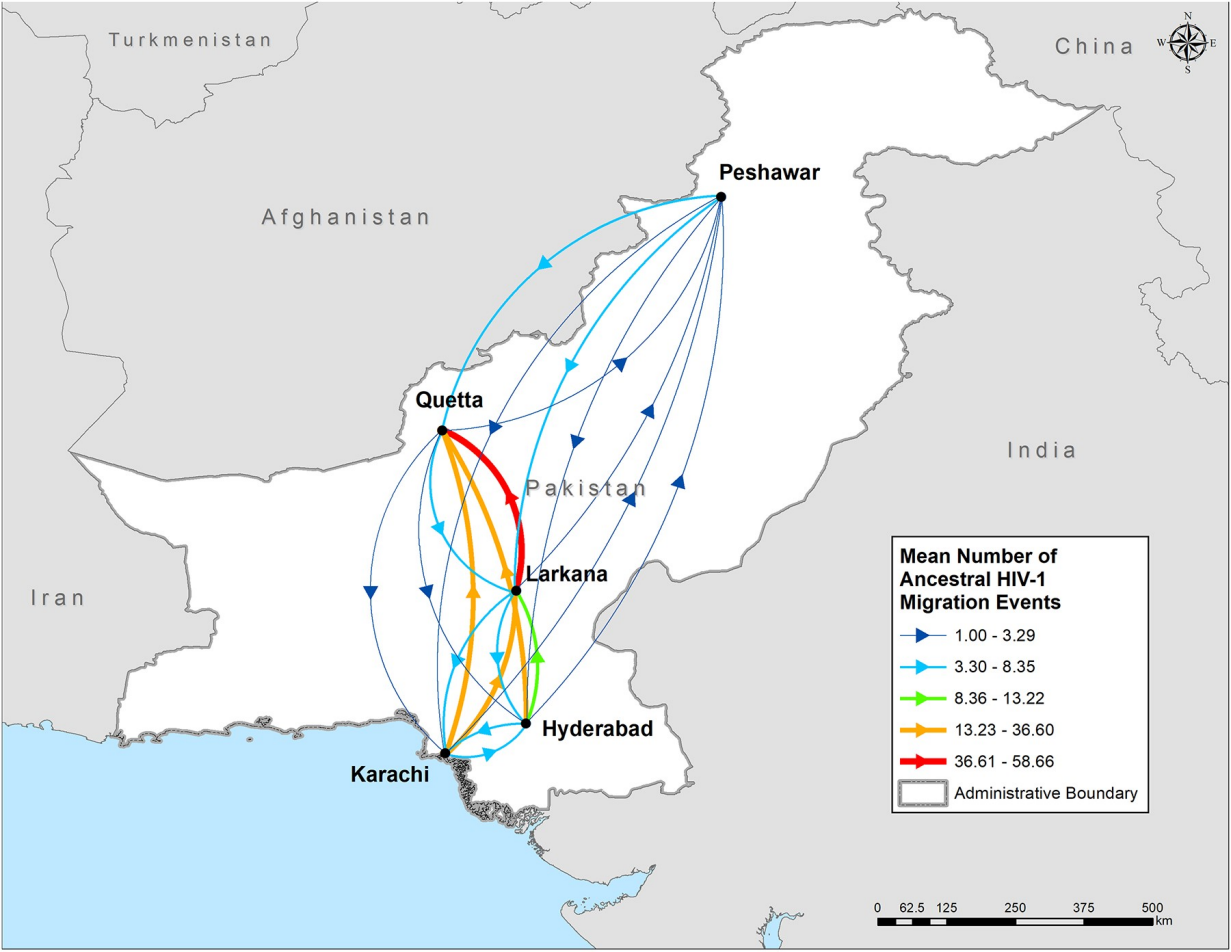
Spatial dispersion of HIV-1 subtype A1 among people who inject drugs in Pakistan. Arrows between cities represent the number of HIV-1 migration events inferred using a MCMC structured coalescent approach with MultiTypeTree as implemented in BEAST2. Arrow colors reflect the number of HIV-1 migration events between cities according to the legend at the bottom right corner. This map was created using ESRI ArcMap software version 10.5., using open source data from the Global Administrative Areas version 2.8 (https://gadm.org/data.html).

A structured coalescent based analysis was undertaken in order to investigate the spatial relationship of HIV-1 transmission among the various cities under study. The analysis was limited to subtype A1 sequences since it represents the prevalent strain of HIV-1 circulating among PWID in Pakistan. Inferred HIV-1 migrations between cities can be found in Table 2 and Fig 5 while the relevant maximum clade credibility (MCC) tree is provided in S1 Fig. In general, the highest number of southward HIV-1 migrations events were observed from Quetta to Larkana (5.18 ± 3.85 HIV-1 migration events) and Peshawar to Quetta (6.84 ± 3.86 HIV-1 migration events) while the highest northward migrations rates were observed from Karachi to Larkana (36.60 ± 24.08 HIV-1 migration events) and Larkana to Quetta (58.66 ± 24.78 HIV-1 migration events). Although, the majority of the ancestral nodes were poorly supported according to the MCC tree (posterior probability <0.70, S1 Fig). This was most likely due to polytomies as a result of a high degree of sequence clustering. Time to the most common recent ancestor was relatively short (approximately 1.5 years) and Quetta was identified as the most probable ancestral node location.

**Table 2:** Inferred HIV-1 subtype A1 migration between cities.

Migration direction	Mean number of ancestral migration events (events [n =] from city *x* to city *y*)	Mean number of ancestral migration events standard deviation (± events [n =] from city *x* to city *y*)
Hyderabad → Karachi	4.32	5.32
Hyderabad → Larkana	13.22	16.14
Hyderabad → Peshawar	1.00	1.27
Hyderabad → Quetta	25.67	16.86
Karachi → Hyderabad	8.35	11.02
Karachi → Larkana	36.60	24.08
Karachi → Peshawar	1.68	1.63
Karachi → Quetta	24.46	22.12
Larkana → Hyderabad	6.11	7.67
Larkana → Karachi	5.58	6.81
Larkana → Peshawar	1.31	1.51
Larkana → Quetta	58.66	24.78
Peshawar → Hyderabad	2.64	2.55
Peshawar → Karachi	2.80	2.57
Peshawar → Larkana	4.17	3.26
Peshawar → Quetta	6.84	3.86
Quetta → Hyderabad	3.29	3.13
Quetta → Karachi	3.15	2.99
Quetta → Larkana	5.62	5.15
Quetta → Peshawar	1.16	1.33

## Discussion

Pakistan is currently facing a concentrated HIV-1 epidemic among PWID in multiple cities including Karachi, Hyderabad, Larkana, Quetta and Peshawar despite interventions, such as syringe and needle exchange programs [1]. Earlier phylogenetic studies reported that the HIV-1 epidemic may have been the result of the transmission of a founding subtype A1 virus originating in the 1990s [7]. Our study indicates that this is no longer the case since we observed greater subtype heterogeneity among HIV-1 positive PWID especially in cities such as Karachi. Although subtype A1 is still the dominant circulating strain in Pakistan, greater subtype heterogeneity may be indicative of overlapping sexual and/or drug injecting networks between PWID and other high-risk populations. Existing behavioural data [13] suggest significant sexual and/or drug injection networking between PWID and female sex workers (FSW), hijra sex workers (HSW), and male sex workers (MSW) as well as Afghan refugees residing in Pakistan [14, 15]. Phylogenetic analysis of HIV-1 *gag* gene sequences [14] showed that the HIV-1 epidemic in Pakistani MSM is represented by HIV-1 subtypes A, G, CRF01_AE and CRF02_AG while these subtypes in addition to CRF35_AD can be found circulating among HIV-1 positive Afghan refugees living in Karachi [15]. It would not be unexpected that extremely high risk injecting reported earlier [5, 13], in an environment of increasing HIV-1 prevalence, could contribute to frequent infection of a single individual with multiple subtypes of HIV-1 leading to the generation of unique recombinant forms. A unique method of assisted injection, locally referred to as “scale”, was first reported among PWID in the city of Sargodha in 2009 [3, 5]. This practice involves collecting blood-drug mixtures (“scale”) as payment for professional injection services by double-pumping syringes. The “scale” is then either sold to another client or used by the injector. Recently, Chen *et al*. [10] reported on unique CRF02_AG/A1 recombinants, indicating ongoing recombination between locally transmitted CRF02_AG and A1 strains, suggesting that these unique recombinants may become major epidemic strains in Pakistan. It is likely that we underestimated the proportion of recombinant HIV-1 sequences in our population given we only sequenced a portion of the HIV-1 *pol* gene. More extensive, whole genome-based sequence analysis would be required to resolve the potential HIV-1 recombination events among Pakistani PWID.

Drug injecting behaviours vary between cities, however unsafe injection practices, large injection networks and the use of professional injectors are common among PWID in Pakistan [5]. Therefore, it is not surprising that nearly three quarters of the HIV-1 *pol* sequences from this study are incorporated into HIV-1 phylogenetic transmission clusters. Seeking help injecting drugs from professional injectors has previously been identified as a strong correlate of HIV-1 infection [5]. At the time of DBS collection, professional injectors were being employed as peer support workers in a number of the cities involved in this study (personal communications with the National AIDS Control Program). Our observation that the odds of clustering was 10.1% less among individuals who regularly sought assistance in injecting drugs supports the utility of this intervention at limiting onward HIV-1 transmission among PWID (manuscript in preparation). Consistent with this observation, one of the largest clusters we identified (n = 52) mainly consisted of individuals sampled in Peshawar (51 of 52) who did not seek help injecting drugs from professional injectors. At present, it is unclear if professional injectors have been employed as peer support workers in Peshawar, but our results would suggest that there may be other unidentified epidemic drivers in this particular city. Alternatively, it may indicate that some study participants did not understand the concept of sharing, did not understand questions pertaining to high risk injecting behaviors, or were unwilling to admit participation in such behaviors.

Currently, Afghanistan produces approximately 80% of the world’s heroin supply [30]. Most of the heroin is trafficked through Pakistan before reaching its final destination in Europe, the Russian Federation and Asia [4]. As such, local consumption markets have been established mainly positioned along important regional trafficking routes. Once inside Pakistan, a significant amount of heroin is trafficked south along main roads and railways southward to Karachi [4]. Concurrently, acetic anhydride–a crucial precursor chemical for the synthesis of opium into heroin–is trafficked into Afghanistan allegedly along the same trafficking routes, albeit in the northward direction [16]. Acetic anhydride importation is prohibited in Afghanistan therefore the precursor needs to be smuggled into the country–primarily via Pakistan. Even though the earliest documented cases of HIV-1 infection were reported in Karachi [17], the first outbreak reported among PWID was observed approximately 500 km to the North in Larkana [18]. At the time, Shah *et al*. (2004) noted an increase in injection drug use and indicated that Larkana had become a “hub” of drug use activities, attracting users from all parts of the country. Shortly thereafter, (≤ 2 years) the epidemic spread to multiple cities, solidifying Pakistan’s concentrated epidemic among PWID (www.nacp.gov.pk/whatwedo/surveillance.html) [2, 19]. The relatively short time to the most common recent ancestor observed in our analysis is consistent with rapid rises in HIV-1 infection among PWID in Pakistan. Taken altogether, the flow of opioid trafficking and epidemiological data are consistent with the observed HIV-1 migration patterns based on our molecular findings that place Larkana at the center of the PWID HIV-1 epidemic in Pakistan. Yet, we interpret these results with caution since most ancestral nodes had low posterior probability values. Several cities facing a concentrated HIV-1 epidemic [1] were not sampled during this study, so HIV-1 migration patterns could change with the addition of new/different sampling locations. Furthermore, it would also be important to consider internal population displacement (IDP) due to economic hardship, natural disasters and conflict (http://hrcp-web.org/hrcpweb/wp-content/pdf/ff/22.pdf) [20] as possible explanations for the observed migration patterns.

Initially, high quality powder heroin was abundant in Pakistan with smoking being the primary means of consumption [20]. However, recent conflict in neighboring Afghanistan may have directly or indirectly facilitated transitions to injection drug use [20] in Pakistan by causing fluctuations in prices, purity and availability of heroin. It is widely known that the Taliban relies on heroin production and trafficking to fund operations. Consistent with this, trends in opium cultivation over the last decade have generally reflected conflict intensity in the area [21]. Based on our Bayesian skyline plot analysis, changes in HIV-1 effective population size estimates among PWID in Pakistan correspond with fluctuations in opium cultivation in Afghanistan [12]. More specifically, both spikes in estimates of HIV-1 effective population sizes coincided with increases in opium poppy cultivation. This observation suggests that the transmission of HIV-1 among PWID in Pakistan is associated with local trends in opium trafficking–influenced by conflict–and exacerbated by unsafe injection practices. Nevertheless, our analysis was limited to sequences from samples collected in 2005, 2007 and 2014 so we cannot rule out other possibilities that could explain instances of HIV-1 effective population growth like contractions of HIV/AIDS programs caused by the reallocation of funds to disaster relief efforts (i.e. major flooding) over the last decade [22].

## Conclusions

In summary, our results suggest that the current epidemic among PWID is no longer an onward transmission of a limited number of A1 subtype founder viruses as reported previously. The greater subtype heterogeneity would be consistent with sexual and/or drug injecting networks between PWID and other most-at-risk populations reported by others. Although it is evident that unsafe injection behaviors played a significant role in driving the rise in HIV-1 prevalence among PWID, local trends in illicit opioid trafficking may have influenced injection behaviors and facilitated HIV-1 transmission as a result. Based on both epidemiologic and molecular findings, the HIV-1 epidemic does not appear to have simply spread northward from Karachi but that Larkana may have had a more significant role in the amplifying of the PWID HIV-1 epidemic. Since the transmission of HIV-1 between PWID appears to be associated with trends in drug trafficking, it is possible that intimate knowledge of the drug trade may help anticipate future HIV-1 outbreaks. It will also be interesting to compare the phylogenetic data described above with that collected from other urban centers to determine if our observations are consistent in other PWID communities throughout Pakistan.

## Materials and methods

### Study participants and biological sample collection

PWID were recruited from the Pakistani cities of Karachi (n = 300), Larkana (n = 300), Peshawar (n = 253), Quetta (n = 300) and Hyderabad (n = 300) from August to December, 2014. A detailed mapping of size, geography and operation typology of the PWID population was undertaken prior to the actual survey to ensure that the collected sample accurately reflects the study population. Mapping methodology is described in detail elsewhere [23]. Field teams recruited study participants at PWID hotspots using multiple techniques such as multi-stage cluster sampling, snowball sampling, and time location cluster sampling [24]. After obtaining informed consent, participants were interviewed using a structured questionnaire to collect information pertaining to sociodemographics, drug use practices, social networks, sexual behavior, and utilization of services for HIV testing and care. A summary of the baseline sociodemographic characteristics of the study population is provided in Table 3. Participants were also asked if they were currently receiving antiretrovirals (ARVs). Among those who answered (n = 49), only 4 individuals indicated that they were receiving ARVs. At the completion of the interview, DBS specimens were collected by the standard finger prick method using a self-retracting safety lancet device. After saturating each of the five spots printed on Whatman 903 filter paper cards (GE Healthcare Life Sciences, Mississauga, ON), DBS were air dried for a minimum of 3 hours at ambient temperature prior to storage at room temperature in sealed gas-impermeable bags containing desiccant pouches [25].

**Table 3:** Baseline sociodemographic characteristics of PWID recruited from the Pakistani cities of Karachi, Larkana, Peshawar, Quetta and Hyderabad.

	HIV-1 negative	HIV-1 positive
	N = 1,086	N = 367
	% (N)	% (N)
Age category		
20–24	18.8 (204)	24.0 (88)
25–29	26.6 (289)	33.2 (122)
30–34	18.5 (201)	18.8 (69)
35–39	13.9 (151)	9.3 (34)
≧40	19.3 (210)	9.8 (36)
<20	2.9 (31)	4.9 (18)
Gender identity		
Female	0.6 (7)	0.8 (3)
Male	99.0 (1,075)	99.2 (364)
Transgender	0.4 (4)	0 (0)
Years of education		
1–6 years	25.1 (268)	24.8 (90)
≧7 years	21.6 (231)	22.9 (83)
None	45.9 (490)	43.8 (159)
Quranic	7.4 (79)	8.5 (31)
Marital status		
Married	35.2 (379)	32.1 (117)
Seperated/divorced/widowed	16.2 (174)	11.2 (41)
Unmarried	48.7 (524)	56.7 (207)
Slept in their own home		
No	31.5 (342)	30.3 (111)
Yes	68.5 (744)	69.8 (256)

### Ethics statement

Ethical approval was obtained from the Health Research Ethics Board at the University of Manitoba [HS15691(H2012:294)], Canada and BRIDGE Consultants Foundation, Pakistan. Informed consent was obtained verbally from study participants for the behavioural survey and biological sampling components of this study. Verbal consent was chosen due to varying levels of literacy among study participants and as an additional measure of protecting their identities and documented by a member of the data collection team.

### Serological testing

All DBS specimen cards were screened for HIV-1 with the AVIOQ HIV-1 Microelisa System (Avioq Inc., Durham, NC) according to the manufacturer’s instructions.

### Isolation of nucleic acids, amplification and sequencing

A routine, in-house HIV-1 drug resistance mutation genotyping assay was used to sequence a portion of the *pol* open reading frame (nt 2,074–3,334 on HXB2, K03455). Internal validation (results not shown) determined that the lower limit of detection of the assay on DBS is approximately ≥1,000 HIV-1 copies/mL. Briefly, total nucleic acid was isolated from a single DBS (approximately 75 μL of whole blood) using an automated, magnetic silica-based, NucliSENS easyMAG instrument (bioMérieux, St-Laurent, QC) according to the manufacturer’s specific B protocol (version 2.0.1). Each DBS were lysed for 1 hour at room temperature with gentle agitation in 2 mL of NucliSENS lysis buffer (bioMérieux). Purified nucleic acid was eluted in 50 μL of NucliSENS extraction buffer 3 (bioMérieux) and stored immediately at -80°C. The *protease* (PROT) and partial *reverse transcriptase* (RT) genes were amplified in two separate fragments by reverse transcriptase PCR (S3–S5 Tables) followed by nested PCR (S6–S8 Tables) with the help of the SuperScript III one-step RT-PCR system (ThermoFisher Scientific, Burlington, ON) and AmpliTaq Gold DNA polymerase (ThermoFisher Scientific) respectively. Sanger sequencing was performed using the BigDye Terminator v3.1 cycle sequencing kit (ThermoFisher Scientific) with nested PCR and sequencing primers (S9 Table). Sequence analysis and contig assembly were performed using RECall to standardize sequence interpretation [26]. We also included PWID HIV-1 *pol* sequences from Karachi (accession no. JQ011747, JQ011748, and JQ011750—JQ011780) and Sargodha (accession no. JQ011625—JQ011628, JQ011630, JQ011632, JQ011634, JQ011637, JQ011639, JQ011641, JQ011642, JQ011652, JQ011654, JQ011655, JQ011656, JQ011662, JQ011663, JQ011664, JQ011670, JQ011673, JQ011675, JQ011676, JQ011678, JQ011680, JQ011681, JQ011687, JQ011688, JQ011690, JQ011691, JQ011695, JQ011699, JQ011700, JQ011702, JQ011704, JQ011709, JQ011710, JQ011714, JQ011720, JQ011721, JQ011726, JQ011729, JQ011732, JQ011733, JQ011742, and JQ011743) reported during outbreaks in 2005 and 2007, respectively.

### Subtyping and phylogenetic cluster analysis

PWID HIV-1 *pol* sequences were aligned, visually inspected and manually edited in MEGA v7 [27] as required. Codons associated with major drug-resistance mutations were not removed from sequence alignments because participants were most likely antiretroviral treatment (ART)-naïve due to poor coverage of HIV/AIDS treatment programs. [28] Each PWID specimen was subtyped with the REGA HIV-1 automated subtyping tool v3 [29] and COMET [30]. In the event of discordant results, SCUEAL [31] was used as a tiebreaker.

For phylogenetic cluster analysis, reference and background sequences were obtained from the Los Alamos HIV database (http://www.hiv.lanl.gov/). Furthermore, the top 10 BLASTn hits for each PWID HIV-1 sequence were also included as background sequences. Duplicate sequences were removed from the alignment. Maximum likelihood (ML) trees were reconstructed with IQ-TREE v1.4.4 [32], using the best-fitting substitution model selected automatically by IQ-TREE. Reliability of the tree topologies was assessed by ultra-fast bootstrap [33] re-sampling (100,000 replicates). Monophyletic clades were identified with ClusterPicker v1.2.3 using 90% bootstrap support, a genetic distance cutoff of 4.5% and a large cluster threshold of 5 [34]. Trees were visualized and annotated in FigTree v1.4.3 (http://tree.bio.ed.ac.uk/software/figtree/). Odds of PWID sequences clustering were calculated using odds ratios in Epi Info for Windows v7 (https://www.cdc.gov/epiinfo/index.html).

Phylogenetic clusters were inferred using a second method based on tip-to-tip (patristic) distance between sequences measured on phylogenetic trees [35]. Briefly, HIV-1 pol sequences from all participants were aligned using MAFFT v7.2.2.1 [36], visually inspected using AliView v.1.15 [37], and codons associated with major drug-resistance mutations were removed. A distribution of 100 phylogenies was inferred using a General Time Reversible (GTR) nucleotide substitution model and FastTree2 v2.1.8 [38]. Phylogenetic clusters of 5 or more participants consistent across all 100 trees were identified using a tip-to-tip (patristic) distance cutoff of <0.02 substitutions per site. Clusters were visualized and annotated in Cytoscape v3.7.2 (https://cytoscape.org/).

### Phylodynamic analysis

Effective population size estimates (Bayesian skyline plot analysis) were inferred using a Bayesian Markov Chain Monte Carlo (MCMC) analysis of time-stamped taxa using BEAST v1.10.4 [39] under a general time reversible (GTR) substitution model (4 gamma categories), a strict molecular clock model set to a rate of 2.5 x 10^−3^ substitutions/site/year (1.1, 4.0; 95% confidence interval) [40], a coalescent Bayesian Skyline tree prior [41] and chain lengths of 1–9 x 10^8^ sampled to generate 10,000 trees. Different demographic models were compared by marginal likelihood estimation (i.e. path sampling/stepping-stone sampling) as implemented in BEAST v1.10.4 [42]. The coalescent Bayesian Skyline tree prior had the highest log marginal likelihood (-13,303.7) compared to the coalescent constant size (-13,500.2), coalescent exponential size (-13,360.6), coalescent logistic growth (-13,351.6), and coalescent Bayesian SkyGrid (-13,386.0) tree priors. Convergence of each run was assessed using Tracer v1.7.1 to ensure that effective sample sizes (ESS) >200 were obtained for all statistics and that acceptable mixing was achieved [43]. Effective population size estimates of HIV-1 were limited to subtype A1 sequences from this study and databases sequences (listed above) with injection drug use as a known risk behavior. HIV-1 subtype A1 sequences were chosen according to the subtyping approach described above. Any potential recombinant HIV-1 subtype A1 sequences were identified with Recombination Detection Program (RDP) v4.97 [44] and omitted from HIV-1 effective population size estimates (S10 Table). The relationship between effective HIV-1 population size estimates and opium cultivation was modeled by linear regression in SPSS (IBM, Armonk, NY).

HIV-1 migration was inferred using a MCMC structured coalescent approach with MultiTypeTree [45] as implemented in BEAST2 v2.4.5 [46] under a GTR substitution model (4 gamma categories), a strict molecular clock model set to a rate of 1.52 x 10^−3^ substitutions/site/year with a lognormal distribution, and chain lengths >5 x 10^8^ sampled to generate 10,000 trees. The strict molecular clock rate represents the posterior from effective population size estimates described above. HIV-1 migration is measured in terms of the number of ancestral migrations events from city *x* to city *y*. Restrictions were set on population size (log_10_) for each city based on mapping exercises carried out in Pakistan [1]. Convergence of each run was assessed using Tracer 1.7.1 to ensure that ESS >200 were obtained for all statistics and that acceptable mixing was achieved [43]. Structured coalescent analysis was limited to HIV-1 subtype A1 sequences from our study given the computational limitations for reliable ancestral migration estimates [45]. Older database sequences (e.g. from Karachi and Sargodha) were omitted from this analysis to provide a contemporary snapshot of HIV-1 migration. PWID in Pakistan are a highly mobile population [1] therefore, including older database sequences may not be representative of the most current HIV-1 migration patterns. HIV-1 subtype A1 sequences were chosen according to the subtyping approach described above. Any potential recombinant HIV-1 subtype A1 sequences were identified with RDP v4.97 [44] and omitted from the structured coalescent analysis (S10 Table). All XML files are available upon request.

## Supporting information

S1 Fig The maximum clade credibility (MCC) tree, summarizing the posterior tree distribution from the MultiTypeTree analysis. Nodes have been annotated with the type posterior probability (below) and associated dates (above). Only support values greater than 70 are displayed. The 95% highest posterior density (HPD) intervals of the dates associated to the root and select nodes are located within parentheses. The type posterior probability is defined as the posterior probability of the most probable location. Years are displayed as labels on the x-axis.
(TIFF)

S1 Table HIV-1 subtype assignment. (DOCX)

S2 Table HIV-1 sequences included in the phylogenetic cluster analysis. (DOCX)

S3 Table Description of RT-PCR primers for in-house HIV-1 drug resistance mutation genotyping. (DOCX)

S4 Table Master mix preparation for the reverse transcription and subsequent amplification (RT-PCR) of HIV-1 PROT and partial RT genes. (DOCX)

S5 Table Thermal cycling conditions for RT-PCR. (DOCX)

S6 Table Description of nested PCR primers for in-house HIV-1 drug resistance mutation genotyping. (DOCX)

S7 Table Master mix preparation for the second round (nested PCR) of HIV-1 PROT and partial RT gene amplification. (DOCX)

S8 Table Thermal cycling conditions for nested PCR. (DOCX)

S9 Table Additional HIV-1 PROT and partial RT sequencing primers. (DOCX)

S10 Table Potential recombinant HIV-1 subtype A1 sequences identified with RDP v4.97. (DOCX)
