# Supplementary material for: HIV-1 phylodynamic analysis among people who inject drugs in Pakistan correlates with trends in illicit opioid trade
Source: PLoS One. 2020 Aug 28;15(8):e0237560. doi: 10.1371/journal.pone.0237560 (PMC7454939; doi:10.1371/journal.pone.0237560)
Supplement: S1 Table — (DOCX) [file pone.0237560.s002.docx]

| Study ID | Accession no. | REGA | COMET | SCUEAL | Subtype |
| --- | --- | --- | --- | --- | --- |
| PWID.HYD.0006.2014 | MN887780 | A (A1) | A1 | - | A1 |
| PWID.HYD.0010.2014 | MN888029 | A (A1) | A1 | - | A1 |
| PWID.HYD.0020.2014 | MN888030 | A (A1) | A1 | - | A1 |
| PWID.HYD.0035.2014 | MN888031 | A (A1) | A1 | - | A1 |
| PWID.HYD.0056.2014 | MN888032 | A (A1) | A1 | - | A1 |
| PWID.HYD.0062.2014 | MN888033 | A (A1) | A1 | - | A1 |
| PWID.HYD.0064.2014 | MN888034 | A (A1) | A1 | - | A1 |
| PWID.HYD.0069.2014 | MN888035 | A (A1) | A1 | - | A1 |
| PWID.HYD.0083.2014 | MN888036 | A (A1) | A1 | - | A1 |
| PWID.HYD.0085.2014 | MN888037 | A (A1) | A1 | - | A1 |
| PWID.HYD.0093.2014 | MN888038 | A (A1) | A1 | - | A1 |
| PWID.HYD.0096.2014 | MN888039 | A (A1) | A1 | - | A1 |
| PWID.HYD.0097.2014 | MN888040 | A (A1) | A1 | - | A1 |
| PWID.HYD.0099.2014 | MN888041 | A (A1) | A1 | - | A1 |
| PWID.HYD.0106.2014 | MN888042 | A (A1) | A1 | - | A1 |
| PWID.HYD.0117.2014 | MN888043 | A (A1) | A1 | - | A1 |
| PWID.HYD.0125.2014 | MN888044 | A (A1) | A1 | - | A1 |
| PWID.HYD.0133.2014 | MN888045 | A (A1) | A1 | - | A1 |
| PWID.HYD.0134.2014 | MN888046 | G (02_AG) | G (check for 02_AG) | - | CRF02_AG |
| PWID.HYD.0146.2014 | MN888047 | A (A1) | A1 | - | A1 |
| PWID.HYD.0157.2014 | MN888048 | A (35_AD) | A1 (check for 35_AD) | A1 | A1 |
| PWID.HYD.0161.2014 | MN888049 | CRF 02_AG | G (check for 02_AG) | - | CRF02_AG |
| PWID.HYD.0166.2014 | MN888050 | A (A1) | A1 | - | A1 |
| PWID.HYD.0167.2014 | MN888051 | A (A1) | A1 | - | A1 |
| PWID.HYD.0169.2014 | MN888052 | A (A1) | A1 | - | A1 |
| PWID.HYD.0173.2014 | MN888053 | A (A1) | A1 | - | A1 |
| PWID.HYD.0174.2014 | MN888054 | A (A1) | A1 | - | A1 |
| PWID.HYD.0182.2014 | MN888055 | Recombinant of G, A1 | Unassigned_2, "02_AG-A1" | A1 | Recombinant/unassigned |
| PWID.HYD.0184.2014 | MN888056 | A (A1) | A1 | - | A1 |
| PWID.HYD.0195.2014 | MN888057 | A (A1) | A1 | - | A1 |
| PWID.HYD.0200.2014 | MN888058 | A (A1) | A1 | - | A1 |
| PWID.HYD.0217.2014 | MN888059 | A (A1) | A1 | - | A1 |
| PWID.HYD.0218.2014 | MN888060 | A (A1) | A1 | - | A1 |
| PWID.HYD.0243.2014 | MN888061 | A (A1) | A1 | - | A1 |
| PWID.HYD.0245.2014 | MN888062 | A (A1) | A1 | - | A1 |
| PWID.HYD.0246.2014 | MN888063 | A (A1) | A1 | - | A1 |
| PWID.HYD.0250.2014 | MN888064 | A (A1) | A1 | - | A1 |
| PWID.HYD.0257.2014 | MN888065 | A (A1) | A1 | - | A1 |
| PWID.HYD.0266.2014 | MN888066 | A (A1) | A1 | - | A1 |
| PWID.HYD.0272.2014 | MN888067 | A (A1) | A1 | - | A1 |
| PWID.HYD.0277.2014 | MN888068 | A (A1) | A1 | - | A1 |
| PWID.HYD.0289.2014 | MN888069 | A (A1) | A1 | - | A1 |
| PWID.KAR.0001.2014 | MN887781 | A (A1) | A1 | - | A1 |
| PWID.KAR.0003.2014 | MN887782 | CRF 02_AG | G (check for 02_AG) | - | CRF02_AG |
| PWID.KAR.0013.2014 | MN887783 | A (A1) | A1 | - | A1 |
| PWID.KAR.0017.2014 | MN887784 | A (A1) | A1 | - | A1 |
| PWID.KAR.0026.2014 | MN887785 | A (A1) | A1 | - | A1 |
| PWID.KAR.0027.2014 | MN887786 | A (A1) | A1 | - | A1 |
| PWID.KAR.0028.2014 | MN887787 | A (A1) | A1 | - | A1 |
| PWID.KAR.0029.2014 | MN887788 | A (A1) | A1 | - | A1 |
| PWID.KAR.0032.2014 | MN887789 | A (A1) | A1 | - | A1 |
| PWID.KAR.0034.2014 | MN887790 | A (A1) | A1 | - | A1 |
| PWID.KAR.0035.2014 | MN887791 | A (A1) | A1 | - | A1 |
| PWID.KAR.0036.2014 | MN887792 | A (A1) | A1 | - | A1 |
| PWID.KAR.0037.2014 | MN887793 | A (A1) | A1 | - | A1 |
| PWID.KAR.0038.2014 | MN887794 | A (A1) | A1 | - | A1 |
| PWID.KAR.0040.2014 | MN887795 | A (A1) | A1 | - | A1 |
| PWID.KAR.0041.2014 | MN887796 | A (A1) | A1 | - | A1 |
| PWID.KAR.0042.2014 | MN887797 | A (A1) | A1 | - | A1 |
| PWID.KAR.0051.2014 | MN887798 | A (A1) | A1 | - | A1 |
| PWID.KAR.0056.2014 | MN887799 | A (A1) | A1 | - | A1 |
| PWID.KAR.0059.2014 | MN887800 | A (A1) | A1 | - | A1 |
| PWID.KAR.0061.2014 | MN887801 | A (A1) | A1 | - | A1 |
| PWID.KAR.0067.2014 | MN887802 | A (A1) | A1 | - | A1 |
| PWID.KAR.0070.2014 | MN887803 | A (A1) | A1 | - | A1 |
| PWID.KAR.0072.2014 | MN887804 | A (A1) | A1 | - | A1 |
| PWID.KAR.0075.2014 | MN887805 | A (A1) | A1 | - | A1 |
| PWID.KAR.0076.2014 | MN887806 | A (A1) | A1 | - | A1 |
| PWID.KAR.0078.2014 | MN887807 | C | C | - | C |
| PWID.KAR.0079.2014 | MN887808 | A (A1) | A1 (check for 35_AD) | CRF35-like | CRF35_AD |
| PWID.KAR.0081.2014 | MN887809 | A (A1) | A1 | - | A1 |
| PWID.KAR.0083.2014 | MN887810 | A (A1) | A1 | - | A1 |
| PWID.KAR.0084.2014 | MN887811 | A (A1) | A1 | - | A1 |
| PWID.KAR.0085.2014 | MN887812 | A (A1) | A1 | - | A1 |
| PWID.KAR.0088.2014 | MN887813 | A (A1) | A1 | - | A1 |
| PWID.KAR.0089.2014 | MN887814 | A (A1) | A1 | - | A1 |
| PWID.KAR.0091.2014 | MN887815 | A (A1) | A1 | - | A1 |
| PWID.KAR.0097.2014 | MN887816 | CRF 35_AD | A1 (check for 35_AD) | CRF35-like | CRF35_AD |
| PWID.KAR.0104.2014 | MN887817 | CRF 35_AD | A1 (check for 35_AD) | CRF35-like | CRF35_AD |
| PWID.KAR.0105.2014 | MN887818 | A (A1) | A1 | - | A1 |
| PWID.KAR.0108.2014 | MN887819 | G (02_AG) | 02_AG | - | CRF02_AG |
| PWID.KAR.0114.2014 | MN887820 | A (A1) | A1 | - | A1 |
| PWID.KAR.0130.2014 | MN887821 | G (02_AG) | 02_AG | - | CRF02_AG |
| PWID.KAR.0131.2014 | MN887822 | G (02_AG) | 02_AG | - | CRF02_AG |
| PWID.KAR.0135.2014 | MN887823 | G (02_AG) | 02_AG | - | CRF02_AG |
| PWID.KAR.0139.2014 | MN887824 | G (02_AG) | 02_AG | - | CRF02_AG |
| PWID.KAR.0140.2014 | MN887825 | G (02_AG) | 02_AG | - | CRF02_AG |
| PWID.KAR.0141.2014 | MN887826 | G (02_AG) | 02_AG | - | CRF02_AG |
| PWID.KAR.0143.2014 | MN887827 | A (A1) | A1 | - | A1 |
| PWID.KAR.0144.2014 | MN887828 | G (02_AG) | 02_AG | - | CRF02_AG |
| PWID.KAR.0145.2014 | MN887829 | G (02_AG) | 02_AG | - | CRF02_AG |
| PWID.KAR.0147.2014 | MN887830 | G (02_AG) | 02_AG | - | CRF02_AG |
| PWID.KAR.0149.2014 | MN887831 | CRF 02_AG | 02_AG | - | CRF02_AG |
| PWID.KAR.0150.2014 | MN887832 | CRF 02_AG | 02_AG | - | CRF02_AG |
| PWID.KAR.0151.2014 | MN887833 | CRF 02_AG | 02_AG | - | CRF02_AG |
| PWID.KAR.0153.2014 | MN887834 | CRF 02_AG | 02_AG | - | CRF02_AG |
| PWID.KAR.0154.2014 | MN887835 | Recombinant of A1, G | 02_AG | A1 | Recombinant/unassigned |
| PWID.KAR.0155.2014 | MN887836 | A (35_AD) | A1 (check for 35_AD) | - | CRF35_AD |
| PWID.KAR.0156.2014 | MN887837 | A (A1) | A1 | - | A1 |
| PWID.KAR.0160.2014 | MN887838 | G (02_AG) | 02_AG | - | CRF02_AG |
| PWID.KAR.0163.2014 | MN887839 | CRF 02_AG | 02_AG | - | CRF02_AG |
| PWID.KAR.0167.2014 | MN887840 | A (A1) | A1 | - | A1 |
| PWID.KAR.0170.2014 | MN887841 | G (02_AG) | 02_AG | - | CRF02_AG |
| PWID.KAR.0171.2014 | MN887842 | CRF 02_AG | A1 (check for 02_AG) | - | CRF02_AG |
| PWID.KAR.0172.2014 | MN887843 | CRF 02_AG | A1 (check for 02_AG) | - | CRF02_AG |
| PWID.KAR.0173.2014 | MN887844 | A (A1), potential recombinant | A1 (check for 02_AG) | G | Recombinant/unassigned |
| PWID.KAR.0180.2014 | MN887845 | A (A1) | A1 | - | A1 |
| PWID.KAR.0183.2014 | MN887846 | A (A1) | A1 | - | A1 |
| PWID.KAR.0188.2014 | MN887847 | A (A1) | A1 | - | A1 |
| PWID.KAR.0190.2014 | MN887848 | A (A1) | A1 | - | A1 |
| PWID.KAR.0191.2014 | MN887849 | A (A1) | A1 | - | A1 |
| PWID.KAR.0192.2014 | MN887850 | Recombinant of A1, G | A1 | A1 | A1 |
| PWID.KAR.0196.2014 | MN887851 | A (A1) | A1 | - | A1 |
| PWID.KAR.0197.2014 | MN887852 | A (A1) | A1 | - | A1 |
| PWID.KAR.0198.2014 | MN887853 | G (02_AG) | 02_AG | - | CRF02_AG |
| PWID.KAR.0201.2014 | MN887854 | CRF 35_AD | A1 (check for 35_AD) | - | CRF35_AD |
| PWID.KAR.0202.2014 | MN887855 | A (A1) | A1 | - | A1 |
| PWID.KAR.0206.2014 | MN887856 | CRF 02_AG | 02_AG | - | CRF02_AG |
| PWID.KAR.0207.2014 | MN887857 | A (A1) | A1 | - | A1 |
| PWID.KAR.0209.2014 | MN887858 | A (A1) | A1 | - | A1 |
| PWID.KAR.0214.2014 | MN887859 | A (A1) | A1 | - | A1 |
| PWID.KAR.0231.2014 | MN887860 | G | 02_AG | A | Recombinant/unassigned |
| PWID.KAR.0233.2014 | MN887861 | A (A1) | A1 | - | A1 |
| PWID.KAR.0234.2014 | MN887862 | A (A1) | A1 | - | A1 |
| PWID.KAR.0235.2014 | MN887863 | A (A1) | A1 | - | A1 |
| PWID.KAR.0236.2014 | MN887864 | Recombinant of A1, G | A1 | Complex | Recombinant/unassigned |
| PWID.KAR.0238.2014 | MN887865 | A (A1) | A1 | - | A1 |
| PWID.KAR.0242.2014 | MN887866 | A (A1) | A1 | - | A1 |
| PWID.KAR.0243.2014 | MN887867 | A (A1) | A1 | - | A1 |
| PWID.KAR.0246.2014 | MN887868 | A (A1) | A1 | - | A1 |
| PWID.KAR.0248.2014 | MN887869 | A (A1) | A1 | - | A1 |
| PWID.KAR.0249.2014 | MN887870 | G (02_AG) | 02_AG | - | CRF02_AG |
| PWID.KAR.0250.2014 | MN887871 | Recombinant of A1, G | A1 | Complex | Recombinant/unassigned |
| PWID.KAR.0251.2014 | MN887872 | A (A1) | A1 | - | A1 |
| PWID.KAR.0252.2014 | MN887873 | A (A1) | A1 | - | A1 |
| PWID.KAR.0253.2014 | MN887874 | Recombinant of A1, G | A1 | Complex | Recombinant/unassigned |
| PWID.KAR.0254.2014 | MN887875 | A (A1) | A1 | - | A1 |
| PWID.KAR.0256.2014 | MN887876 | A (A1) | A1 | - | A1 |
| PWID.KAR.0259.2014 | MN887877 | A (A1) | A1 | - | A1 |
| PWID.KAR.0260.2014 | MN887878 | G (02_AG) | 02_AG | - | CRF02_AG |
| PWID.KAR.0261.2014 | MN887879 | Recombinant of A1, G | A1 (check for 02_AG) | Complex | Recombinant/unassigned |
| PWID.KAR.0264.2014 | MN887880 | A (A1) | A1 | - | A1 |
| PWID.KAR.0266.2014 | MN887881 | A (A1) | A1 | - | A1 |
| PWID.KAR.0267.2014 | MN887882 | G (02_AG) | 02_AG | - | CRF02_AG |
| PWID.KAR.0271.2014 | MN887883 | A (A1) | A1 | - | A1 |
| PWID.KAR.0273.2014 | MN887884 | Recombinant of A1, G | A1 | Complex | Recombinant/unassigned |
| PWID.KAR.0275.2014 | MN887885 | A (A1) | A1 | - | A1 |
| PWID.KAR.0292.2014 | MN887886 | G (02_AG) | 02_AG | - | CRF02_AG |
| PWID.KAR.0294.2014 | MN887887 | A (A1) | A1 | - | A1 |
| PWID.KAR.0299.2014 | MN887888 | A (A1) | A1 | - | A1 |
| PWID.KAR.0300.2014 | MN887889 | G (02_AG) | 02_AG | - | CRF02_AG |
| PWID.LAR.0005.2014 | MN887890 | CRF 02_AG | G (check for 02_AG) | - | CRF02_AG |
| PWID.LAR.0009.2014 | MN887891 | A (A1) | A1 | - | A1 |
| PWID.LAR.0014.2014 | MN887892 | A (A1) | A1 | - | A1 |
| PWID.LAR.0015.2014 | MN887893 | Recombinant of 02_AG, A1 | G (check for 02_AG) | G | G |
| PWID.LAR.0016.2014 | MN887894 | A (A1) | A1 | - | A1 |
| PWID.LAR.0029.2014 | MN887895 | A (A1) | A1 | - | A1 |
| PWID.LAR.0033.2014 | MN887896 | A (A1) | A1 | - | A1 |
| PWID.LAR.0042.2014 | MN887897 | A (A1) | A1 | - | A1 |
| PWID.LAR.0051.2014 | MN887898 | A (A1) | A1 | - | A1 |
| PWID.LAR.0052.2014 | MN887899 | A (A1) | A1 | - | A1 |
| PWID.LAR.0058.2014 | MN887900 | A (A1) | A1 | - | A1 |
| PWID.LAR.0190.2014 | MN887901 | A (A1) | A1 | - | A1 |
| PWID.LAR.0193.2014 | MN887902 | A (A1) | A1 | - | A1 |
| PWID.LAR.0194.2014 | MN887903 | A (A1) | A1 | - | A1 |
| PWID.LAR.0203.2014 | MN887904 | A (A1) | A1 | - | A1 |
| PWID.LAR.0206.2014 | MN887905 | G (02_AG) | G (check for 02_AG) | G | G |
| PWID.LAR.0231.2014 | MN887906 | A (A1) | A1 | - | A1 |
| PWID.LAR.0243.2014 | MN887907 | A (A1) | A1 | - | A1 |
| PWID.LAR.0249.2014 | MN887908 | A (A1) | A1 | - | A1 |
| PWID.LAR.0256.2014 | MN887909 | A (A1) | A1 | - | A1 |
| PWID.LAR.0277.2014 | MN887910 | A (A1) | A1 | - | A1 |
| PWID.LAR.0278.2014 | MN887911 | A (A1) | A1 | - | A1 |
| PWID.LAR.0295.2014 | MN887912 | A (A1) | A1 | - | A1 |
| PWID.LAR.0299.2014 | MN887913 | A (A1) | A1 | - | A1 |
| PWID.LAR.0312.2014 | MN887914 | A (A1) | A1 | - | A1 |
| PWID.PES.0001.2014 | MN887915 | A (A1) | A1 | - | A1 |
| PWID.PES.0002.2014 | MN887916 | A (A1) | A1 | - | A1 |
| PWID.PES.0004.2014 | MN887917 | Recombinant of A1, G | A1 (check for 02_AG) | A | Recombinant/unassigned |
| PWID.PES.0005.2014 | MN887918 | A (A1) | A1 | - | A1 |
| PWID.PES.0011.2014 | MN887919 | A (A1) | A1 | - | A1 |
| PWID.PES.0015.2014 | MN887920 | A (A1) | A1 | - | A1 |
| PWID.PES.0018.2014 | MN887921 | A (A1) | A1 | - | A1 |
| PWID.PES.0019.2014 | MN887922 | A (A1) | A1 | - | A1 |
| PWID.PES.0020.2014 | MN887923 | A (A1) | A1 | - | A1 |
| PWID.PES.0023.2014 | MN887924 | A (A1) | A1 | - | A1 |
| PWID.PES.0025.2014 | MN887925 | A (A1) | A1 | - | A1 |
| PWID.PES.0027.2014 | MN887926 | A (A1) | A1 | - | A1 |
| PWID.PES.0031.2014 | MN887927 | A (A1) | A1 | - | A1 |
| PWID.PES.0033.2014 | MN887928 | A (A1) | A1 | - | A1 |
| PWID.PES.0035.2014 | MN887929 | A (A1) | A1 | - | A1 |
| PWID.PES.0036.2014 | MN887930 | A (A1) | A1 | - | A1 |
| PWID.PES.0038.2014 | MN887931 | A (A1) | A1 | - | A1 |
| PWID.PES.0039.2014 | MN887932 | A (A1) | A1 | - | A1 |
| PWID.PES.0040.2014 | MN887933 | A (A1) | A1 | - | A1 |
| PWID.PES.0041.2014 | MN887934 | A (A1) | A1 | - | A1 |
| PWID.PES.0042.2014 | MN887935 | Recombinant of A1, G | A1 | Complex | Recombinant/unassigned |
| PWID.PES.0043.2014 | MN887936 | A (A1) | unassigned_1, A1-D | A1 | A1 |
| PWID.PES.0044.2014 | MN887937 | A (A1) | A1 | - | A1 |
| PWID.PES.0045.2014 | MN887938 | A (A1) | A1 | - | A1 |
| PWID.PES.0048.2014 | MN887939 | A (A1) | A1 | - | A1 |
| PWID.PES.0053.2014 | MN887940 | A (A1) | A1 | - | A1 |
| PWID.PES.0056.2014 | MN887941 | Recombinant of A1, G | A1 | Complex | Recombinant/unassigned |
| PWID.PES.0057.2014 | MN887942 | A (A1) | A1 | - | A1 |
| PWID.PES.0058.2014 | MN887943 | A (A1) | A1 | - | A1 |
| PWID.PES.0066.2014 | MN887944 | A (A1) | A1 | - | A1 |
| PWID.PES.0068.2014 | MN887945 | A (A1) | A1 | - | A1 |
| PWID.PES.0070.2014 | MN887946 | Recombinant of A1, G | A1 (check for 02_AG) | Complex | Recombinant/unassigned |
| PWID.PES.0072.2014 | MN887947 | A (A1) | A1 | - | A1 |
| PWID.PES.0074.2014 | MN887948 | A (A1) | A1 | - | A1 |
| PWID.PES.0080.2014 | MN887949 | A (A1) | A1 | - | A1 |
| PWID.PES.0083.2014 | MN887950 | A (A1) | A1 | - | A1 |
| PWID.PES.0089.2014 | MN887951 | A (A1) | A1 | - | A1 |
| PWID.PES.0093.2014 | MN887952 | A (A1) | A1 | - | A1 |
| PWID.PES.0095.2014 | MN887953 | A (A1) | A1 | - | A1 |
| PWID.PES.0099.2014 | MN887954 | Recombinant of A1, G | A1 | Complex | Recombinant/unassigned |
| PWID.PES.0100.2014 | MN887955 | A (A1) | A1 | - | A1 |
| PWID.PES.0102.2014 | MN887956 | G (02_AG) | 02_AG | - | CRF02_AG |
| PWID.PES.0109.2014 | MN887957 | A (A1) | A1 | - | A1 |
| PWID.PES.0114.2014 | MN887958 | A (A1) | A1 | - | A1 |
| PWID.PES.0115.2014 | MN887959 | A (A1) | A1 | - | A1 |
| PWID.PES.0117.2014 | MN887960 | Recombinant of A1, G | A1 | Complex | Recombinant/unassigned |
| PWID.PES.0119.2014 | MN887961 | A (A1) | A1 | - | A1 |
| PWID.PES.0123.2014 | MN887962 | A (A1) | A1 | - | A1 |
| PWID.PES.0127.2014 | MN887963 | A (A1) | A1 | - | A1 |
| PWID.PES.0131.2014 | MN887964 | A (A1) | A1 | - | A1 |
| PWID.PES.0132.2014 | MN887965 | A (A1) | A1 | - | A1 |
| PWID.PES.0136.2014 | MN887966 | A (A1) | A1 | - | A1 |
| PWID.PES.0142.2014 | MN887967 | A (A1) | A1 | - | A1 |
| PWID.PES.0144.2014 | MN887968 | G (02_AG) | 02_AG | G | CRF02_AG |
| PWID.PES.0145.2014 | MN887969 | A (A1) | A1 | - | A1 |
| PWID.PES.0148.2014 | MN887970 | A (A1) | A1 | - | A1 |
| PWID.PES.0149.2014 | MN887971 | Recombinant of A1, G | A1 | A1 | A1 |
| PWID.PES.0150.2014 | MN887972 | A (A1) | A1 | - | A1 |
| PWID.PES.0153.2014 | MN887973 | A (A1) | A1 | - | A1 |
| PWID.PES.0154.2014 | MN887974 | A (A1) | A1 | - | A1 |
| PWID.PES.0155.2014 | MN887975 | A (A1) | A1 | - | A1 |
| PWID.PES.0156.2014 | MN887976 | A (A1) | A1 | - | A1 |
| PWID.PES.0157.2014 | MN887977 | A (A1) | A1 | - | A1 |
| PWID.PES.0159.2014 | MN887978 | A (A1) | A1 | - | A1 |
| PWID.PES.0160.2014 | MN887979 | A (A1) | A1 | - | A1 |
| PWID.PES.0161.2014 | MN887980 | Recombinant of A1, G | A1 | Complex | Recombinant/unassigned |
| PWID.PES.0162.2014 | MN887981 | A (A1) | A1 | - | A1 |
| PWID.PES.0163.2014 | MN887982 | A (A1) | A1 | - | A1 |
| PWID.PES.0164.2014 | MN887983 | A (A1) | A1 | - | A1 |
| PWID.PES.0168.2014 | MN887984 | A (A1) | A1 | - | A1 |
| PWID.PES.0169.2014 | MN887985 | A (A1) | A1 | - | A1 |
| PWID.PES.0170.2014 | MN887986 | A (A1) | A1 | - | A1 |
| PWID.PES.0171.2014 | MN887987 | A (A1) | A1 | - | A1 |
| PWID.PES.0177.2014 | MN887988 | A (A1) | A1 | - | A1 |
| PWID.PES.0184.2014 | MN887989 | A (A1) | A1 | - | A1 |
| PWID.PES.0190.2014 | MN887990 | Recombinant of A1, G | A1 | Complex | Recombinant/unassigned |
| PWID.PES.0205.2014 | MN887991 | CRF 02_AG | 02_AG | - | CRF02_AG |
| PWID.PES.0207.2014 | MN887992 | Recombinant of A1, G | A1 | Complex | Recombinant/unassigned |
| PWID.PES.0213.2014 | MN887993 | A (A1) | A1 | - | A1 |
| PWID.PES.0214.2014 | MN887994 | A (A1) | A1 | - | A1 |
| PWID.PES.0217.2014 | MN887995 | A (A1) | A1 | - | A1 |
| PWID.PES.0221.2014 | MN887996 | Recombinant of A1, G | A1 | Complex | Recombinant/unassigned |
| PWID.PES.0232.2014 | MN887997 | A (A1) | A1 | - | A1 |
| PWID.PES.0238.2014 | MN887998 | CRF 02_AG | 02_AG | - | CRF02_AG |
| PWID.PES.0242.2014 | MN887999 | CRF 02_AG | B (check for 02_AG) | - | CRF02_AG |
| PWID.PES.0244.2014 | MN888000 | A (A1) | A1 | - | A1 |
| PWID.PES.0245.2014 | MN888001 | Recombinant of A1, G | A1 | Complex | Recombinant/unassigned |
| PWID.PES.0246.2014 | MN888002 | CRF 02_AG | 02_AG | - | CRF 02_AG |
| PWID.PES.0247.2014 | MN888003 | CRF 02_AG | 02_AG | - | CRF 02_AG |
| PWID.PES.0248.2014 | MN888004 | CRF 02_AG | G (check for 02_AG) | - | CRF 02_AG |
| PWID.PES.0250.2014 | MN888005 | CRF 02_AG | G (check for 02_AG) | - | CRF 02_AG |
| PWID.PES.0251.2014 | MN888006 | CRF 02_AG | A1 (check for 02_AG) | - | CRF02_AG |
| PWID.PES.0256.2014 | MN888007 | Recombinant | A1 (check for 02_AG) | Complex | Recombinant/unassigned |
| PWID.PES.0257.2014 | MN888008 | CRF 02_AG | G (check for 02_AG) | Complex | CRF02_AG |
| PWID.QUE.0004.2014 | MN888009 | A (A1) | A1 | - | A1 |
| PWID.QUE.0008.2014 | MN888010 | A (A1) | A1 | - | A1 |
| PWID.QUE.0018.2014 | MN888011 | A (A1) | A1 | - | A1 |
| PWID.QUE.0031.2014 | MN888012 | A (A1) | A1 | - | A1 |
| PWID.QUE.0035.2014 | MN888013 | A (A1) | A1 | - | A1 |
| PWID.QUE.0064.2014 | MN888014 | G (02_AG) | A1 (check for 02_AG) | G | G |
| PWID.QUE.0106.2014 | MN888015 | A (A1) | A1 | - | A1 |
| PWID.QUE.0111.2014 | MN888016 | A (A1) | A1 (check for 35_AD) | CRF35-like | CRF35_AD |
| PWID.QUE.0120.2014 | MN888017 | A (A1) | A1 | - | A1 |
| PWID.QUE.0121.2014 | MN888018 | A (A1) | A1 | - | A1 |
| PWID.QUE.0151.2014 | MN888019 | A (A1) | A1 | - | A1 |
| PWID.QUE.0173.2014 | MN888020 | A (A1) | A1 | - | A1 |
| PWID.QUE.0176.2014 | MN888021 | A (A1) | A1 | - | A1 |
| PWID.QUE.0180.2014 | MN888022 | A (A1) | A1 | - | A1 |
| PWID.QUE.0191.2014 | MN888023 | A (A1) | A1 | - | A1 |
| PWID.QUE.0222.2014 | MN888024 | A (A1) | A1 | - | A1 |
| PWID.QUE.0231.2014 | MN888025 | A (A1) | A1 | - | A1 |
| PWID.QUE.0232.2014 | MN888026 | A (A1) | A1 | - | A1 |
| PWID.QUE.0253.2014 | MN888027 | A (A1) | A1 | - | A1 |
| PWID.QUE.0262.2014 | MN888028 | A (A1) | A1 | - | A1 |
