# Supplementary material for: HIV-1 phylodynamic analysis among people who inject drugs in Pakistan correlates with trends in illicit opioid trade
Source: PLoS One. 2020 Aug 28;15(8):e0237560. doi: 10.1371/journal.pone.0237560 (PMC7454939; doi:10.1371/journal.pone.0237560)
Supplement: S2 Table — (DOCX) [file pone.0237560.s003.docx]

| AB287378 | Rwanda | NA | 1993 | Database |
| --- | --- | --- | --- | --- |
| AB287379 | Rwanda | NA | 1993 | Database |
| AB703609 | Iran | Kermanshah | 2011 | Database |
| AB703610 | Iran | Shiraz | 2011 | Database |
| AB703615 | Iran | Tehran | 2010 | Database |
| AB716096 | Iran | Tehran | 2010 | Database |
| AB716105 | Iran | Tehran | 2010 | Database |
| AB716107 | Iran | Tehran | 2010 | Database |
| AB716113 | Iran | Tehran | 2010 | Database |
| AB716126 | Iran | Shiraz | 2011 | Database |
| AB716132 | Iran | Tehran | 2010 | Database |
| AB716134 | Iran | Tehran | 2010 | Database |
| AB751432 | Ghana | Koforidua | 2009 | Database |
| AB864091 | Japan | NA | 2007 | Database |
| AF107369 | Uganda | NA | 1991 | Database |
| AF107370 | Uganda | NA | 1991 | Database |
| AF107372 | Uganda | NA | 1991 | Database |
| AF212281 | Liberia | NA | 1994 | Database |
| AF212289 | Ghana | NA | 1994 | Database |
| AF295292 | Italy | NA | 1997 | Database |
| AF295296 | Italy | NA | 1997 | Database |
| AF388133 | Uganda | NA | 1997 | Database |
| AF388151 | Uganda | NA | 1997 | Database |
| AF395347 | USA | NA | NA | Database |
| AF410209 | Uganda | NA | NA | Database |
| AF410211 | Uganda | NA | NA | Database |
| AF410239 | Uganda | NA | NA | Database |
| AF447850 | Somalia | NA | 1989 | Database |
| AF457063 | Kenya | NA | 1999 | Database |
| AF457070 | Kenya | NA | 2000 | Database |
| AJ286936 | Cameroon | NA | 1997 | Database |
| AJ286937 | Cameroon | NA | 1997 | Database |
| AJ286942 | Cameroon | NA | 1997 | Database |
| AJ286943 | Cameroon | NA | 1997 | Database |
| AJ286956 | Cameroon | NA | 1998 | Database |
| AJ286984 | Senegal | NA | 1998 | Database |
| AJ286994 | Senegal | NA | 1998 | Database |
| AJ286996 | Senegal | NA | 1998 | Database |
| AJ287000 | Senegal | NA | 1998 | Database |
| AJ313397 | Gabon | NA | 2000 | Database |
| AJ313402 | Gabon | NA | 2000 | Database |
| AJ583710 | Senegal | NA | 1999 | Database |
| AJ583717 | Senegal | NA | 1999 | Database |
| AJ583724 | Senegal | NA | 1998 | Database |
| AJ583726 | Senegal | NA | 1998 | Database |
| AJ583730 | Senegal | NA | 1999 | Database |
| AM279387 | Cameroon | NA | 1997 | Database |
| AM279388 | Cameroon | NA | 1997 | Database |
| AM706403 | Denmark | NA | 2003 | Database |
| AY017467 | Spain | NA | 1999 | Database |
| AY136960 | SouthAfrica | NA | 2001 | Database |
| AY322184 | Kenya | NA | 1986 | Database |
| AY435227 | Uganda | NA | 1997 | Database |
| AY435245 | Uganda | NA | 1997 | Database |
| AY435365 | Uganda | NA | 1997 | Database |
| AY444181 | Uganda | NA | NA | Database |
| AY444235 | Cameroon | NA | NA | Database |
| AY444255 | Cameroon | NA | NA | Database |
| AY492759 | Kenya | NA | 1997 | Database |
| AY713415 | Somalia | NA | 1989 | Database |
| AY803463 | Uganda | NA | 2003 | Database |
| AY803471 | Uganda | NA | 2003 | Database |
| AY955694 | Uganda | NA | NA | Database |
| AY958334 | Malawi | NA | NA | Database |
| DQ079832 | Uganda | NA | 2002 | Database |
| DQ079834 | Uganda | NA | 2002 | Database |
| EF064537 | Mali | NA | 2005 | Database |
| EF087995 | Cameroon | NA | 2000 | Database |
| EF158040 | Afghanistan | NA | 2005 | Database |
| EF158041 | Afghanistan | NA | 2005 | Database |
| EF158042 | Afghanistan | NA | 2005 | Database |
| EF158043 | Afghanistan | NA | 2005 | Database |
| EF368909 | Canada | NA | NA | Database |
| EF368970 | Canada | NA | NA | Database |
| EF602205 | SouthAfrica | NA | 2002 | Database |
| EF612010 | Uganda | NA | NA | Database |
| EF612077 | Uganda | NA | NA | Database |
| EF612180 | Uganda | NA | NA | Database |
| EU177898 | Ghana | NA | 2002 | Database |
| EU177952 | Ghana | NA | 2002 | Database |
| EU177997 | Ghana | NA | 2002 | Database |
| EU178033 | Ghana | NA | 2002 | Database |
| EU248559 | Belgium | NA | 2003 | Database |
| EU306786 | Uganda | NA | 2006 | Database |
| EU330669 | Mali | NA | 2006 | Database |
| FJ388896 | Cyprus | NA | 2005 | Database |
| FJ577269 | BurkinaFaso | NA | 2005 | Database |
| FJ577271 | BurkinaFaso | NA | 2005 | Database |
| FJ577287 | BurkinaFaso | NA | 2005 | Database |
| FJ577296 | BurkinaFaso | NA | 2006 | Database |
| FJ577306 | BurkinaFaso | NA | 2006 | Database |
| FJ577344 | BurkinaFaso | NA | 2006 | Database |
| FJ577356 | BurkinaFaso | NA | 2006 | Database |
| FJ577366 | BurkinaFaso | NA | 2006 | Database |
| FM955678 | Togo | NA | 2006 | Database |
| FM955697 | Togo | NA | 2006 | Database |
| FN599679 | Senegal | Dakar | 1999 | Database |
| FN599690 | Senegal | Dakar | 1999 | Database |
| FN599708 | Senegal | Dakar | 1999 | Database |
| FN599714 | Senegal | Dakar | 1998 | Database |
| FN599716 | Senegal | Dakar | 1998 | Database |
| GQ398902 | Italy | NA | 2004 | Database |
| GQ399266 | Italy | NA | 2005 | Database |
| GQ409580 | Uganda | NA | 2005 | Database |
| GQ462429 | United Kingdom | NA | NA | Database |
| GQ477442 | Afghanistan | NA | 2006 | Database |
| GQ477444 | Afghanistan | NA | 206 | Database |
| GQ477445 | Afghanistan | NA | 2006 | Database |
| GQ477446 | Afghanistan | NA | 2007 | Database |
| GQ477449 | Afghanistan | NA | 2007 | Database |
| GQ477451 | Afghanistan | NA | 2006 | Database |
| GU207180 | BurkinaFaso | NA | 2003 | Database |
| GU207209 | BurkinaFaso | NA | 2003 | Database |
| GU724810 | Iran | NA | 2009 | Database |
| GU724815 | Iran | NA | 2009 | Database |
| GU724827 | Iran | NA | 2009 | Database |
| HM002504 | Senegal | NA | 2009 | Database |
| HQ170625 | Ghana | NA | 2007 | Database |
| JN620499 | Pakistan | NA | 2009 | Database |
| JN620500 | Pakistan | NA | 2009 | Database |
| JN620501 | Pakistan | NA | 2009 | Database |
| JN620502 | Pakistan | NA | 2009 | Database |
| JN620503 | Pakistan | NA | 2009 | Database |
| JN620504 | Pakistan | NA | 2009 | Database |
| JN620505 | Pakistan | NA | 2009 | Database |
| JN620506 | Pakistan | NA | 2009 | Database |
| JN620507 | Pakistan | NA | 2008 | Database |
| JN620508 | Pakistan | NA | 2008 | Database |
| JN620509 | Pakistan | NA | 2008 | Database |
| JN620510 | Pakistan | NA | 2008 | Database |
| JN620511 | Pakistan | NA | 2009 | Database |
| JN620512 | Pakistan | NA | 2009 | Database |
| JN620513 | Pakistan | NA | 2009 | Database |
| JN620514 | Pakistan | NA | 2009 | Database |
| JN620515 | Pakistan | NA | 2009 | Database |
| JN620516 | Pakistan | NA | 2009 | Database |
| JN620517 | Pakistan | NA | 2009 | Database |
| JN620518 | Pakistan | NA | 2009 | Database |
| JN620519 | Pakistan | NA | 2009 | Database |
| JN620520 | Pakistan | NA | 2008 | Database |
| JN620521 | Pakistan | NA | 2009 | Database |
| JN620522 | Pakistan | NA | 2009 | Database |
| JN620523 | Pakistan | NA | 2009 | Database |
| JN620524 | Pakistan | NA | 2009 | Database |
| JN620525 | Pakistan | NA | 2009 | Database |
| JN620526 | Pakistan | NA | 2009 | Database |
| JN620527 | Pakistan | NA | 2009 | Database |
| JN620528 | Pakistan | NA | 2009 | Database |
| JN620529 | Pakistan | NA | 2005 | Database |
| JN620530 | Pakistan | NA | 2005 | Database |
| JN620531 | Pakistan | NA | 2005 | Database |
| JN620532 | Pakistan | NA | 2005 | Database |
| JN620533 | Pakistan | NA | 2005 | Database |
| JN620534 | Pakistan | NA | 2005 | Database |
| JN620535 | Pakistan | NA | 2005 | Database |
| JN620536 | Pakistan | NA | 2005 | Database |
| JN628487 | Kenya | NA | 2009 | Database |
| JN628488 | Kenya | NA | 2009 | Database |
| JN673636 | Senegal | NA | 2009 | Database |
| JQ011625 | Pakistan | Sargodha | 2007 | Database |
| JQ011626 | Pakistan | Sargodha | 2007 | Database |
| JQ011627 | Pakistan | Sargodha | 2007 | Database |
| JQ011628 | Pakistan | Sargodha | 2007 | Database |
| JQ011630 | Pakistan | Sargodha | 2007 | Database |
| JQ011632 | Pakistan | Sargodha | 2007 | Database |
| JQ011634 | Pakistan | Sargodha | 2007 | Database |
| JQ011637 | Pakistan | Sargodha | 2007 | Database |
| JQ011639 | Pakistan | Sargodha | 2007 | Database |
| JQ011641 | Pakistan | Sargodha | 2007 | Database |
| JQ011642 | Pakistan | Sargodha | 2007 | Database |
| JQ011652 | Pakistan | Sargodha | 2007 | Database |
| JQ011654 | Pakistan | Sargodha | 2007 | Database |
| JQ011655 | Pakistan | Sargodha | 2007 | Database |
| JQ011656 | Pakistan | Sargodha | 2007 | Database |
| JQ011662 | Pakistan | Sargodha | 2007 | Database |
| JQ011663 | Pakistan | Sargodha | 2007 | Database |
| JQ011664 | Pakistan | Sargodha | 2007 | Database |
| JQ011670 | Pakistan | Sargodha | 2007 | Database |
| JQ011673 | Pakistan | Sargodha | 2007 | Database |
| JQ011675 | Pakistan | Sargodha | 2007 | Database |
| JQ011676 | Pakistan | Sargodha | 2007 | Database |
| JQ011678 | Pakistan | Sargodha | 2007 | Database |
| JQ011680 | Pakistan | Sargodha | 2007 | Database |
| JQ011681 | Pakistan | Sargodha | 2007 | Database |
| JQ011687 | Pakistan | Sargodha | 2007 | Database |
| JQ011688 | Pakistan | Sargodha | 2007 | Database |
| JQ011690 | Pakistan | Sargodha | 2007 | Database |
| JQ011691 | Pakistan | Sargodha | 2007 | Database |
| JQ011695 | Pakistan | Sargodha | 2007 | Database |
| JQ011699 | Pakistan | Sargodha | 2007 | Database |
| JQ011700 | Pakistan | Sargodha | 2007 | Database |
| JQ011702 | Pakistan | Sargodha | 2007 | Database |
| JQ011704 | Pakistan | Sargodha | 2007 | Database |
| JQ011709 | Pakistan | Sargodha | 2007 | Database |
| JQ011710 | Pakistan | Sargodha | 2007 | Database |
| JQ011714 | Pakistan | Sargodha | 2007 | Database |
| JQ011720 | Pakistan | Sargodha | 2007 | Database |
| JQ011721 | Pakistan | Sargodha | 2007 | Database |
| JQ011726 | Pakistan | Sargodha | 2007 | Database |
| JQ011729 | Pakistan | Sargodha | 2007 | Database |
| JQ011732 | Pakistan | Sargodha | 2007 | Database |
| JQ011733 | Pakistan | Sargodha | 2007 | Database |
| JQ011742 | Pakistan | Sargodha | 2007 | Database |
| JQ011743 | Pakistan | Sargodha | 2007 | Database |
| JQ011747 | Pakistan | Karachi | 2005 | Database |
| JQ011748 | Pakistan | Karachi | 2005 | Database |
| JQ011749 | Pakistan | Karachi | 2005 | Database |
| JQ011750 | Pakistan | Karachi | 2005 | Database |
| JQ011751 | Pakistan | Karachi | 2005 | Database |
| JQ011752 | Pakistan | Karachi | 2005 | Database |
| JQ011753 | Pakistan | Karachi | 2005 | Database |
| JQ011754 | Pakistan | Karachi | 2005 | Database |
| JQ011755 | Pakistan | Karachi | 2005 | Database |
| JQ011756 | Pakistan | Karachi | 2005 | Database |
| JQ011757 | Pakistan | Karachi | 2005 | Database |
| JQ011758 | Pakistan | Karachi | 2005 | Database |
| JQ011759 | Pakistan | Karachi | 2005 | Database |
| JQ011760 | Pakistan | Karachi | 2005 | Database |
| JQ011761 | Pakistan | Karachi | 2005 | Database |
| JQ011762 | Pakistan | Karachi | 2005 | Database |
| JQ011763 | Pakistan | Karachi | 2005 | Database |
| JQ011764 | Pakistan | Karachi | 2005 | Database |
| JQ011765 | Pakistan | Karachi | 2005 | Database |
| JQ011766 | Pakistan | Karachi | 2005 | Database |
| JQ011767 | Pakistan | Karachi | 2005 | Database |
| JQ011768 | Pakistan | Karachi | 2005 | Database |
| JQ011769 | Pakistan | Karachi | 2005 | Database |
| JQ011770 | Pakistan | Karachi | 2005 | Database |
| JQ011771 | Pakistan | Karachi | 2005 | Database |
| JQ011772 | Pakistan | Karachi | 2005 | Database |
| JQ011773 | Pakistan | Karachi | 2005 | Database |
| JQ011774 | Pakistan | Karachi | 2005 | Database |
| JQ011776 | Pakistan | Karachi | 2005 | Database |
| JQ011777 | Pakistan | Karachi | 2005 | Database |
| JQ011779 | Pakistan | Karachi | 2005 | Database |
| JQ011780 | Pakistan | Karachi | 2005 | Database |
| JQ364518 | Rwanda | NA | 2005 | Database |
| JQ364522 | Rwanda | NA | 2005 | Database |
| JQ364524 | Rwanda | NA | 2005 | Database |
| JQ364526 | Rwanda | NA | 2005 | Database |
| JQ364528 | Rwanda | NA | 2005 | Database |
| JQ364532 | Rwanda | NA | 2005 | Database |
| JX428569 | Spain | NA | 2009 | Database |
| JX460107 | USA | NA | 2008 | Database |
| JX460205 | USA | NA | 2009 | Database |
| KC018523 | SouthAfrica | NA | 2006 | Database |
| KC513713 | Rwanda | NA | 2005 | Database |
| KC513715 | Rwanda | NA | 2005 | Database |
| KC513716 | Rwanda | NA | 2005 | Database |
| KC513717 | Rwanda | NA | 2005 | Database |
| KC516923 | Kenya | NA | 2006 | Database |
| KC516998 | Kenya | NA | 2006 | Database |
| KF531026 | Tanzania | NA | 2005 | Database |
| KF531030 | Tanzania | NA | 2005 | Database |
| KF531032 | Tanzania | NA | 2005 | Database |
| KF531471 | Tanzania | NA | 2005 | Database |
| KF531483 | Tanzania | NA | 2005 | Database |
| KF544043 | Iran | NA | 2011 | Database |
| KF544048 | Iran | NA | 2011 | Database |
| KJ906969 | Uganda | NA | 2007 | Database |
| KJ907030 | Uganda | NA | 2007 | Database |
| KJ907161 | Uganda | NA | 2009 | Database |
| KJ907178 | Uganda | NA | 2009 | Database |
| KM050646 | Zambia | NA | 2000 | Database |
| KR860851 | Botswana | NA | 2008 | Database |
| KR861233 | Botswana | NA | 2008 | Database |
| KT737002 | SouthAfrica | NA | 2006 | Database |
| KU498341 | UnitedKingdom | NA | 2008 | Database |
| KU498457 | UnitedKingdom | NA | 2008 | Database |
| KX465357 | Germany | NA | 2002 | Database |
| KX692024 | Switzerland | NA | 1995 | Database |
| MN887780 | Pakistan | Hyderabad | 2014 | This study |
| MN888029 | Pakistan | Hyderabad | 2014 | This study |
| MN888030 | Pakistan | Hyderabad | 2014 | This study |
| MN888031 | Pakistan | Hyderabad | 2014 | This study |
| MN888032 | Pakistan | Hyderabad | 2014 | This study |
| MN888033 | Pakistan | Hyderabad | 2014 | This study |
| MN888034 | Pakistan | Hyderabad | 2014 | This study |
| MN888035 | Pakistan | Hyderabad | 2014 | This study |
| MN888036 | Pakistan | Hyderabad | 2014 | This study |
| MN888037 | Pakistan | Hyderabad | 2014 | This study |
| MN888038 | Pakistan | Hyderabad | 2014 | This study |
| MN888039 | Pakistan | Hyderabad | 2014 | This study |
| MN888040 | Pakistan | Hyderabad | 2014 | This study |
| MN888041 | Pakistan | Hyderabad | 2014 | This study |
| MN888042 | Pakistan | Hyderabad | 2014 | This study |
| MN888043 | Pakistan | Hyderabad | 2014 | This study |
| MN888044 | Pakistan | Hyderabad | 2014 | This study |
| MN888045 | Pakistan | Hyderabad | 2014 | This study |
| MN888046 | Pakistan | Hyderabad | 2014 | This study |
| MN888047 | Pakistan | Hyderabad | 2014 | This study |
| MN888048 | Pakistan | Hyderabad | 2014 | This study |
| MN888049 | Pakistan | Hyderabad | 2014 | This study |
| MN888050 | Pakistan | Hyderabad | 2014 | This study |
| MN888051 | Pakistan | Hyderabad | 2014 | This study |
| MN888052 | Pakistan | Hyderabad | 2014 | This study |
| MN888053 | Pakistan | Hyderabad | 2014 | This study |
| MN888054 | Pakistan | Hyderabad | 2014 | This study |
| MN888055 | Pakistan | Hyderabad | 2014 | This study |
| MN888056 | Pakistan | Hyderabad | 2014 | This study |
| MN888057 | Pakistan | Hyderabad | 2014 | This study |
| MN888058 | Pakistan | Hyderabad | 2014 | This study |
| MN888059 | Pakistan | Hyderabad | 2014 | This study |
| MN888060 | Pakistan | Hyderabad | 2014 | This study |
| MN888061 | Pakistan | Hyderabad | 2014 | This study |
| MN888062 | Pakistan | Hyderabad | 2014 | This study |
| MN888063 | Pakistan | Hyderabad | 2014 | This study |
| MN888064 | Pakistan | Hyderabad | 2014 | This study |
| MN888065 | Pakistan | Hyderabad | 2014 | This study |
| MN888066 | Pakistan | Hyderabad | 2014 | This study |
| MN888067 | Pakistan | Hyderabad | 2014 | This study |
| MN888068 | Pakistan | Hyderabad | 2014 | This study |
| MN888069 | Pakistan | Hyderabad | 2014 | This study |
| MN887781 | Pakistan | Karachi | 2014 | This study |
| MN887782 | Pakistan | Karachi | 2014 | This study |
| MN887783 | Pakistan | Karachi | 2014 | This study |
| MN887784 | Pakistan | Karachi | 2014 | This study |
| MN887785 | Pakistan | Karachi | 2014 | This study |
| MN887786 | Pakistan | Karachi | 2014 | This study |
| MN887787 | Pakistan | Karachi | 2014 | This study |
| MN887788 | Pakistan | Karachi | 2014 | This study |
| MN887789 | Pakistan | Karachi | 2014 | This study |
| MN887790 | Pakistan | Karachi | 2014 | This study |
| MN887791 | Pakistan | Karachi | 2014 | This study |
| MN887792 | Pakistan | Karachi | 2014 | This study |
| MN887793 | Pakistan | Karachi | 2014 | This study |
| MN887794 | Pakistan | Karachi | 2014 | This study |
| MN887795 | Pakistan | Karachi | 2014 | This study |
| MN887796 | Pakistan | Karachi | 2014 | This study |
| MN887797 | Pakistan | Karachi | 2014 | This study |
| MN887798 | Pakistan | Karachi | 2014 | This study |
| MN887799 | Pakistan | Karachi | 2014 | This study |
| MN887800 | Pakistan | Karachi | 2014 | This study |
| MN887801 | Pakistan | Karachi | 2014 | This study |
| MN887802 | Pakistan | Karachi | 2014 | This study |
| MN887803 | Pakistan | Karachi | 2014 | This study |
| MN887804 | Pakistan | Karachi | 2014 | This study |
| MN887805 | Pakistan | Karachi | 2014 | This study |
| MN887806 | Pakistan | Karachi | 2014 | This study |
| MN887807 | Pakistan | Karachi | 2014 | This study |
| MN887808 | Pakistan | Karachi | 2014 | This study |
| MN887809 | Pakistan | Karachi | 2014 | This study |
| MN887810 | Pakistan | Karachi | 2014 | This study |
| MN887811 | Pakistan | Karachi | 2014 | This study |
| MN887812 | Pakistan | Karachi | 2014 | This study |
| MN887813 | Pakistan | Karachi | 2014 | This study |
| MN887814 | Pakistan | Karachi | 2014 | This study |
| MN887815 | Pakistan | Karachi | 2014 | This study |
| MN887816 | Pakistan | Karachi | 2014 | This study |
| MN887817 | Pakistan | Karachi | 2014 | This study |
| MN887818 | Pakistan | Karachi | 2014 | This study |
| MN887819 | Pakistan | Karachi | 2014 | This study |
| MN887820 | Pakistan | Karachi | 2014 | This study |
| MN887821 | Pakistan | Karachi | 2014 | This study |
| MN887822 | Pakistan | Karachi | 2014 | This study |
| MN887823 | Pakistan | Karachi | 2014 | This study |
| MN887824 | Pakistan | Karachi | 2014 | This study |
| MN887825 | Pakistan | Karachi | 2014 | This study |
| MN887826 | Pakistan | Karachi | 2014 | This study |
| MN887827 | Pakistan | Karachi | 2014 | This study |
| MN887828 | Pakistan | Karachi | 2014 | This study |
| MN887829 | Pakistan | Karachi | 2014 | This study |
| MN887830 | Pakistan | Karachi | 2014 | This study |
| MN887831 | Pakistan | Karachi | 2014 | This study |
| MN887832 | Pakistan | Karachi | 2014 | This study |
| MN887833 | Pakistan | Karachi | 2014 | This study |
| MN887834 | Pakistan | Karachi | 2014 | This study |
| MN887835 | Pakistan | Karachi | 2014 | This study |
| MN887836 | Pakistan | Karachi | 2014 | This study |
| MN887837 | Pakistan | Karachi | 2014 | This study |
| MN887838 | Pakistan | Karachi | 2014 | This study |
| MN887839 | Pakistan | Karachi | 2014 | This study |
| MN887840 | Pakistan | Karachi | 2014 | This study |
| MN887841 | Pakistan | Karachi | 2014 | This study |
| MN887842 | Pakistan | Karachi | 2014 | This study |
| MN887843 | Pakistan | Karachi | 2014 | This study |
| MN887844 | Pakistan | Karachi | 2014 | This study |
| MN887845 | Pakistan | Karachi | 2014 | This study |
| MN887846 | Pakistan | Karachi | 2014 | This study |
| MN887847 | Pakistan | Karachi | 2014 | This study |
| MN887848 | Pakistan | Karachi | 2014 | This study |
| MN887849 | Pakistan | Karachi | 2014 | This study |
| MN887850 | Pakistan | Karachi | 2014 | This study |
| MN887851 | Pakistan | Karachi | 2014 | This study |
| MN887852 | Pakistan | Karachi | 2014 | This study |
| MN887853 | Pakistan | Karachi | 2014 | This study |
| MN887854 | Pakistan | Karachi | 2014 | This study |
| MN887855 | Pakistan | Karachi | 2014 | This study |
| MN887856 | Pakistan | Karachi | 2014 | This study |
| MN887857 | Pakistan | Karachi | 2014 | This study |
| MN887858 | Pakistan | Karachi | 2014 | This study |
| MN887859 | Pakistan | Karachi | 2014 | This study |
| MN887860 | Pakistan | Karachi | 2014 | This study |
| MN887861 | Pakistan | Karachi | 2014 | This study |
| MN887862 | Pakistan | Karachi | 2014 | This study |
| MN887863 | Pakistan | Karachi | 2014 | This study |
| MN887864 | Pakistan | Karachi | 2014 | This study |
| MN887865 | Pakistan | Karachi | 2014 | This study |
| MN887866 | Pakistan | Karachi | 2014 | This study |
| MN887867 | Pakistan | Karachi | 2014 | This study |
| MN887868 | Pakistan | Karachi | 2014 | This study |
| MN887869 | Pakistan | Karachi | 2014 | This study |
| MN887870 | Pakistan | Karachi | 2014 | This study |
| MN887871 | Pakistan | Karachi | 2014 | This study |
| MN887872 | Pakistan | Karachi | 2014 | This study |
| MN887873 | Pakistan | Karachi | 2014 | This study |
| MN887874 | Pakistan | Karachi | 2014 | This study |
| MN887875 | Pakistan | Karachi | 2014 | This study |
| MN887876 | Pakistan | Karachi | 2014 | This study |
| MN887877 | Pakistan | Karachi | 2014 | This study |
| MN887878 | Pakistan | Karachi | 2014 | This study |
| MN887879 | Pakistan | Karachi | 2014 | This study |
| MN887880 | Pakistan | Karachi | 2014 | This study |
| MN887881 | Pakistan | Karachi | 2014 | This study |
| MN887882 | Pakistan | Karachi | 2014 | This study |
| MN887883 | Pakistan | Karachi | 2014 | This study |
| MN887884 | Pakistan | Karachi | 2014 | This study |
| MN887885 | Pakistan | Karachi | 2014 | This study |
| MN887886 | Pakistan | Karachi | 2014 | This study |
| MN887887 | Pakistan | Karachi | 2014 | This study |
| MN887888 | Pakistan | Karachi | 2014 | This study |
| MN887889 | Pakistan | Karachi | 2014 | This study |
| MN887890 | Pakistan | Larkana | 2014 | This study |
| MN887891 | Pakistan | Larkana | 2014 | This study |
| MN887892 | Pakistan | Larkana | 2014 | This study |
| MN887893 | Pakistan | Larkana | 2014 | This study |
| MN887894 | Pakistan | Larkana | 2014 | This study |
| MN887895 | Pakistan | Larkana | 2014 | This study |
| MN887896 | Pakistan | Larkana | 2014 | This study |
| MN887897 | Pakistan | Larkana | 2014 | This study |
| MN887898 | Pakistan | Larkana | 2014 | This study |
| MN887899 | Pakistan | Larkana | 2014 | This study |
| MN887900 | Pakistan | Larkana | 2014 | This study |
| MN887901 | Pakistan | Larkana | 2014 | This study |
| MN887902 | Pakistan | Larkana | 2014 | This study |
| MN887903 | Pakistan | Larkana | 2014 | This study |
| MN887904 | Pakistan | Larkana | 2014 | This study |
| MN887905 | Pakistan | Larkana | 2014 | This study |
| MN887906 | Pakistan | Larkana | 2014 | This study |
| MN887907 | Pakistan | Larkana | 2014 | This study |
| MN887908 | Pakistan | Larkana | 2014 | This study |
| MN887909 | Pakistan | Larkana | 2014 | This study |
| MN887910 | Pakistan | Larkana | 2014 | This study |
| MN887911 | Pakistan | Larkana | 2014 | This study |
| MN887912 | Pakistan | Larkana | 2014 | This study |
| MN887913 | Pakistan | Larkana | 2014 | This study |
| MN887914 | Pakistan | Larkana | 2014 | This study |
| MN887915 | Pakistan | Peshawar | 2014 | This study |
| MN887916 | Pakistan | Peshawar | 2014 | This study |
| MN887917 | Pakistan | Peshawar | 2014 | This study |
| MN887918 | Pakistan | Peshawar | 2014 | This study |
| MN887919 | Pakistan | Peshawar | 2014 | This study |
| MN887920 | Pakistan | Peshawar | 2014 | This study |
| MN887921 | Pakistan | Peshawar | 2014 | This study |
| MN887922 | Pakistan | Peshawar | 2014 | This study |
| MN887923 | Pakistan | Peshawar | 2014 | This study |
| MN887924 | Pakistan | Peshawar | 2014 | This study |
| MN887925 | Pakistan | Peshawar | 2014 | This study |
| MN887926 | Pakistan | Peshawar | 2014 | This study |
| MN887927 | Pakistan | Peshawar | 2014 | This study |
| MN887928 | Pakistan | Peshawar | 2014 | This study |
| MN887929 | Pakistan | Peshawar | 2014 | This study |
| MN887930 | Pakistan | Peshawar | 2014 | This study |
| MN887931 | Pakistan | Peshawar | 2014 | This study |
| MN887932 | Pakistan | Peshawar | 2014 | This study |
| MN887933 | Pakistan | Peshawar | 2014 | This study |
| MN887934 | Pakistan | Peshawar | 2014 | This study |
| MN887935 | Pakistan | Peshawar | 2014 | This study |
| MN887936 | Pakistan | Peshawar | 2014 | This study |
| MN887937 | Pakistan | Peshawar | 2014 | This study |
| MN887938 | Pakistan | Peshawar | 2014 | This study |
| MN887939 | Pakistan | Peshawar | 2014 | This study |
| MN887940 | Pakistan | Peshawar | 2014 | This study |
| MN887941 | Pakistan | Peshawar | 2014 | This study |
| MN887942 | Pakistan | Peshawar | 2014 | This study |
| MN887943 | Pakistan | Peshawar | 2014 | This study |
| MN887944 | Pakistan | Peshawar | 2014 | This study |
| MN887945 | Pakistan | Peshawar | 2014 | This study |
| MN887946 | Pakistan | Peshawar | 2014 | This study |
| MN887947 | Pakistan | Peshawar | 2014 | This study |
| MN887948 | Pakistan | Peshawar | 2014 | This study |
| MN887949 | Pakistan | Peshawar | 2014 | This study |
| MN887950 | Pakistan | Peshawar | 2014 | This study |
| MN887951 | Pakistan | Peshawar | 2014 | This study |
| MN887952 | Pakistan | Peshawar | 2014 | This study |
| MN887953 | Pakistan | Peshawar | 2014 | This study |
| MN887954 | Pakistan | Peshawar | 2014 | This study |
| MN887955 | Pakistan | Peshawar | 2014 | This study |
| MN887956 | Pakistan | Peshawar | 2014 | This study |
| MN887957 | Pakistan | Peshawar | 2014 | This study |
| MN887958 | Pakistan | Peshawar | 2014 | This study |
| MN887959 | Pakistan | Peshawar | 2014 | This study |
| MN887960 | Pakistan | Peshawar | 2014 | This study |
| MN887961 | Pakistan | Peshawar | 2014 | This study |
| MN887962 | Pakistan | Peshawar | 2014 | This study |
| MN887963 | Pakistan | Peshawar | 2014 | This study |
| MN887964 | Pakistan | Peshawar | 2014 | This study |
| MN887965 | Pakistan | Peshawar | 2014 | This study |
| MN887966 | Pakistan | Peshawar | 2014 | This study |
| MN887967 | Pakistan | Peshawar | 2014 | This study |
| MN887968 | Pakistan | Peshawar | 2014 | This study |
| MN887969 | Pakistan | Peshawar | 2014 | This study |
| MN887970 | Pakistan | Peshawar | 2014 | This study |
| MN887971 | Pakistan | Peshawar | 2014 | This study |
| MN887972 | Pakistan | Peshawar | 2014 | This study |
| MN887973 | Pakistan | Peshawar | 2014 | This study |
| MN887974 | Pakistan | Peshawar | 2014 | This study |
| MN887975 | Pakistan | Peshawar | 2014 | This study |
| MN887976 | Pakistan | Peshawar | 2014 | This study |
| MN887977 | Pakistan | Peshawar | 2014 | This study |
| MN887978 | Pakistan | Peshawar | 2014 | This study |
| MN887979 | Pakistan | Peshawar | 2014 | This study |
| MN887980 | Pakistan | Peshawar | 2014 | This study |
| MN887981 | Pakistan | Peshawar | 2014 | This study |
| MN887982 | Pakistan | Peshawar | 2014 | This study |
| MN887983 | Pakistan | Peshawar | 2014 | This study |
| MN887984 | Pakistan | Peshawar | 2014 | This study |
| MN887985 | Pakistan | Peshawar | 2014 | This study |
| MN887986 | Pakistan | Peshawar | 2014 | This study |
| MN887987 | Pakistan | Peshawar | 2014 | This study |
| MN887988 | Pakistan | Peshawar | 2014 | This study |
| MN887989 | Pakistan | Peshawar | 2014 | This study |
| MN887990 | Pakistan | Peshawar | 2014 | This study |
| MN887991 | Pakistan | Peshawar | 2014 | This study |
| MN887992 | Pakistan | Peshawar | 2014 | This study |
| MN887993 | Pakistan | Peshawar | 2014 | This study |
| MN887994 | Pakistan | Peshawar | 2014 | This study |
| MN887995 | Pakistan | Peshawar | 2014 | This study |
| MN887996 | Pakistan | Peshawar | 2014 | This study |
| MN887997 | Pakistan | Peshawar | 2014 | This study |
| MN887998 | Pakistan | Peshawar | 2014 | This study |
| MN887999 | Pakistan | Peshawar | 2014 | This study |
| MN888000 | Pakistan | Peshawar | 2014 | This study |
| MN888001 | Pakistan | Peshawar | 2014 | This study |
| MN888002 | Pakistan | Peshawar | 2014 | This study |
| MN888003 | Pakistan | Peshawar | 2014 | This study |
| MN888004 | Pakistan | Peshawar | 2014 | This study |
| MN888005 | Pakistan | Peshawar | 2014 | This study |
| MN888006 | Pakistan | Peshawar | 2014 | This study |
| MN888007 | Pakistan | Peshawar | 2014 | This study |
| MN888008 | Pakistan | Peshawar | 2014 | This study |
| MN888009 | Pakistan | Quetta | 2014 | This study |
| MN888010 | Pakistan | Quetta | 2014 | This study |
| MN888011 | Pakistan | Quetta | 2014 | This study |
| MN888012 | Pakistan | Quetta | 2014 | This study |
| MN888013 | Pakistan | Quetta | 2014 | This study |
| MN888014 | Pakistan | Quetta | 2014 | This study |
| MN888015 | Pakistan | Quetta | 2014 | This study |
| MN888016 | Pakistan | Quetta | 2014 | This study |
| MN888017 | Pakistan | Quetta | 2014 | This study |
| MN888018 | Pakistan | Quetta | 2014 | This study |
| MN888019 | Pakistan | Quetta | 2014 | This study |
| MN888020 | Pakistan | Quetta | 2014 | This study |
| MN888021 | Pakistan | Quetta | 2014 | This study |
| MN888022 | Pakistan | Quetta | 2014 | This study |
| MN888023 | Pakistan | Quetta | 2014 | This study |
| MN888024 | Pakistan | Quetta | 2014 | This study |
| MN888025 | Pakistan | Quetta | 2014 | This study |
| MN888026 | Pakistan | Quetta | 2014 | This study |
| MN888027 | Pakistan | Quetta | 2014 | This study |
| MN888028 | Pakistan | Quetta | 2014 | This study |
