# Supplementary material for: HIV-1 phylodynamic analysis among people who inject drugs in Pakistan correlates with trends in illicit opioid trade
Source: PLoS One. 2020 Aug 28;15(8):e0237560. doi: 10.1371/journal.pone.0237560 (PMC7454939; doi:10.1371/journal.pone.0237560)
Supplement: S3 Table — (DOCX) [file pone.0237560.s004.docx]

| Gene | Position  (HXB2, K03455) | Primer ID | Sequence  (5’ - 3’) | Amplicon length (bp) |
| --- | --- | --- | --- | --- |
| PROT | 2,057-2,085 | 1[1] | TGA ARG AIT GYA CTG ARA GRC AGG CTA AT | ~ 920 |
|  | 2,955-2,979 | 5[1] | AYC TIA TYC CTG GTG TYT CAT TRT T |  |
| RT | 2,706-2,733 | Fb* | GAA AAT CCA TAY AAY ACT CCA RTA TTT G | ~ 700 |
|  | 3,399-3,420 | MJ4[2] | CTG TTA GTG CTT TGG TTC CTC T |  |

* Designed in-house

1. Monleau M, Butel C, Delaporte E, Boillot F, Peeters M. Effect of storage conditions of dried plasma and blood spots on HIV-1 RNA quantification and PCR amplification for drug resistance genotyping. Journal of Antimicrobial Chemotherapy. 2010;65(8):1562-6.

2. France REcherche Nord & Sud Sida-hiv Hépatites. PCR and sequencing procedures: HIV-1 Paris, France 2015 [accessed January 2, 2020]. Available from: <http://www.hivfrenchresistance.org/>.
