# Supplementary material for: HIV-1 phylodynamic analysis among people who inject drugs in Pakistan correlates with trends in illicit opioid trade
Source: PLoS One. 2020 Aug 28;15(8):e0237560. doi: 10.1371/journal.pone.0237560 (PMC7454939; doi:10.1371/journal.pone.0237560)
Supplement: S5 Table — (DOCX) [file pone.0237560.s006.docx]

|  | Step 1 | Step 2 | Step 3 (40 Cycles) | | | | Step 4 | | Step 5 | |
| --- | --- | --- | --- | --- | --- | --- | --- | --- | --- | --- |
| PROT |  |  |  |  |  |  | |  | |  |
| Temperature | 50ºC | 94ºC | 94ºC | 53ºC | 68ºC | 68ºC | | 4ºC | |  |
| Time | 30 min | 2 min | 20 sec | 30 sec | 1.5 min | 5 min | | Hold | |  |
| RT |  |  |  |  |  |  | |  | |  |
| Temperature | 50ºC | 94ºC | 94ºC | 50ºC | 68ºC | 68ºC | | 4ºC | |  |
| Time | 30 min | 2 min | 20 sec | 30 sec | 1 min | 5 min | | Hold | |  |
