# Supplementary material for: HIV-1 phylodynamic analysis among people who inject drugs in Pakistan correlates with trends in illicit opioid trade
Source: PLoS One. 2020 Aug 28;15(8):e0237560. doi: 10.1371/journal.pone.0237560 (PMC7454939; doi:10.1371/journal.pone.0237560)
Supplement: S6 Table — (DOCX) [file pone.0237560.s007.docx]

| Gene | Position  (HXB2, K03455) | Primer ID | Sequence  (5’ - 3’) | Amplicon length (bp) |
| --- | --- | --- | --- | --- |
| PROT | 2074-2095 | H5A* | AGA CAG GCT AAT TTT TTA GGG A | ~ 900 |
|  | 2945-2970 | B(24)[1] | CTG GTG TYT CAT TRT TKR TAC TAG GT |  |
| RT | 2808-2833 | C[1] | TTY TGG GAR GTY CAR YTA GGR ATA CC | ~ 500 |
|  | 3300-3334 | NE135[2] | CCT ACT AAC TTC TGT ATG TCA TTG ACA GTC CAG CT |  |

* Designed in-house

1. World Health Organization. WHO manual for HIV drug resistance testing using dried blood spot specimens. 2010.

2. France REcherche Nord & Sud Sida-hiv Hépatites. PCR and sequencing procedures: HIV-1 Paris, France 2015 [accessed January 2, 2020]. Available from: <http://www.hivfrenchresistance.org/>.
