# Supplementary material for: HIV-1 phylodynamic analysis among people who inject drugs in Pakistan correlates with trends in illicit opioid trade
Source: PLoS One. 2020 Aug 28;15(8):e0237560. doi: 10.1371/journal.pone.0237560 (PMC7454939; doi:10.1371/journal.pone.0237560)
Supplement: S8 Table — (DOCX) [file pone.0237560.s009.docx]

|  | Step 1 | Step 2 (40 Cycles) | | | Step 3 | Step 4 |
| --- | --- | --- | --- | --- | --- | --- |
| PROT |  |  |  |  |  |  |
| Temperature | 95ºC | 94ºC | 53ºC | 72ºC | 72ºC | 4ºC |
| Time | 10 min | 20 sec | 30 sec | 1.5 min | 10 min | Hold |
| RT |  |  |  |  |  |  |
| Temperature | 95ºC | 94ºC | 54ºC | 72ºC | 72ºC | 4ºC |
| Time | 10 min | 20 sec | 30 sec | 1.5 min | 10 min | Hold |
