# Supplementary material for: HIV-1 phylodynamic analysis among people who inject drugs in Pakistan correlates with trends in illicit opioid trade
Source: PLoS One. 2020 Aug 28;15(8):e0237560. doi: 10.1371/journal.pone.0237560 (PMC7454939; doi:10.1371/journal.pone.0237560)
Supplement: S9 Table — (DOCX) [file pone.0237560.s010.docx]

| Position  (HXB2, K03455) | Primer ID | Sequence  (5’ - 3’) |
| --- | --- | --- |
| 2,147-2,165 | F2b(41) | CAG AGC CAR CAG CCC CAC C |
| 2,243-2,266 | E* | CTT TAR CTT CCC TCA GAT CAC TCT |
| 2,813-2,836 | 8* | GGA AGT TCA ATT AGG AAT ACC ACA |
| 2,860-2,885 | G* | AAT CAG TAA CAG TAC TGG ATG TGG GT |
| 2,901-2,925 | F* | TCC TGA AGT CTT YAT CTA AGG GAA C |
| 2,956-2,979 | 7* | ATC TAA TCC CTG GTG TCT CAT TGT |
| 3,231-3,250 | RT4[1] | AGT TCA TAA CCC ATC CAA AG |
| 3,246-3,266 | RT2[2] | CCA TTT ATC AGG ATG GAG TTC |
| 3,304-3,326 | R2[3] | CTT CTG TAT GTC ATT GAC AGT CC |

* Designed in-house

1. France REcherche Nord & Sud Sida-hiv Hépatites. PCR and sequencing procedures: HIV-1 Paris, France 2015 [accessed January 2, 2020]. Available from: <http://www.hivfrenchresistance.org/>.

2. Saravanan S, Vidya M, Balakrishnan P, Kumarasamy N, Solomon SS, Solomon S, et al. Evaluation of two human immunodeficiency virus-1 genotyping systems: ViroSeq 2.0 and an in-house method. Journal of Virological Methods. 2009;159(2):211-6.

3. Zhou Z, Wagar N, DeVos JR, Rottinghaus E, Diallo K, Nguyen DB, et al. Optimization of a low cost and broadly sensitive genotyping assay for HIV-1 drug resistance surveillance and monitoring in resource-limited settings. PLoS ONE. 2011;6 (11):e28184.
