# Supplementary material for: HIV-1 phylodynamic analysis among people who inject drugs in Pakistan correlates with trends in illicit opioid trade
Source: PLoS One. 2020 Aug 28;15(8):e0237560. doi: 10.1371/journal.pone.0237560 (PMC7454939; doi:10.1371/journal.pone.0237560)
Supplement: S10 Table — (DOCX) [file pone.0237560.s011.docx]

| Study ID  (Accession no.) | RDP[1] | GENECONV[2] | BootScan[3] | MaxChi[4] | Chimaera[5] | SiScan[6] | 3Seq[7] |
| --- | --- | --- | --- | --- | --- | --- | --- |
|  | Average p-values | | | | | | |
| PWID.HYD.0182.2014  (MN888055) | 8.52E-05 | 2.64E-02 | 1.60E-03 | 4.59E-04 | 5.68E-04 | 1.42E-04 | 6.53E-07 |
| PWID.KAR.0084.2014  (MN887811) | 1.59E-05 | 3.10E-03 | 2.57E-04 | - | - | 2.95E-04 | 4.30E-05 |
| PWID.KAR.0201.2014  (MN887854) | - | - | - | 5.48E-02 | 1.46E-02 | 3.94E-02 | 3.70E-02 |
| PWID.KAR.0261.2014  (MN887879) | 1.93E-02 | - | 7.07E-03 | 1.64E-02 | 2.95E-04 | 7.60E-07 | 5.50E-06 |
| PWID.PES.0070.2014  (MN887946) | - | - | 4.49E-02 | 2.30E-02 | 2.31E-04 | 7.31E-06 | - |
| PWID.PES.0114.2014  (MN887958) | - | - | - | 7.63E-01 | - | 1.27E-05 | - |
| PWID.PES.0245.2014  (MN888001) | - | - | - | 1.88E-03 | 6.38E-04 | 2.60E-03 | 3.77E-04 |
| PWID.QUE.0111.2014  (MN888016) | - | - | - | 4.20E-02 | 2.26E-02 | 1.94E-02 | - |

1. Martin D, Rybicki E. RDP: detection of recombination amongst aligned sequences. Bioinformatics. 2000;16(6):562-3.

2. Padidam M, Sawyer S, Fauquet CM. Possible emergence of new geminiviruses by frequent recombination. Virology. 1999;265:218-25.

3. Martin DP, Posada D, Crandall KA, Williamson C. A modified bootscan algorithm for automated identification of recombinant sequences and recombination breakpoints. AIDS Research and Human Retroviruses. 2005;21(1):98-102.

4. Smith JM. Analyzing the mosaic structure of genes. Journal of Molecular Evolution. 1992;34:126-9.

5. Posada D, Crandall KA. Evaluation of methods for detecting recombination from DNA sequences: computer simulations. Proceedings of the National Academy of Sciences of the United States of America. 2001;98(24):13757-62.

6. Gibbs MJ, Armstrong JS, Gibbs AJ. Sister-Scanning: a Monte Carlo procedure for assessing signals in recombinant sequences. Bioinformatics. 2000;16(7):573-82.

7. Boni MF, Posada D, Feldman MW. An exact nonparametric method for inferring mosaic structure in sequence triplets. Genetics. 2007;176(2):1035-47.
